# SIRT7 depletion inhibits cell proliferation and androgen-induced autophagy by suppressing the AR signaling in prostate cancer

**DOI:** 10.1186/s13046-019-1516-1

**Published:** 2020-02-04

**Authors:** Mao Ding, Chen-Yi Jiang, Yu Zhang, Jing Zhao, Bang-Min Han, Shu-Jie Xia

**Affiliations:** Department of Urology, Shanghai General Hospital, Shanghai Jiao Tong University School of Medicine, No.100 Haining Road, Hongkou district, Shanghai, 200080 China

**Keywords:** Sirtuin-7, Autophagy, Androgen receptor, Proliferation, Prostate cancer

## Abstract

**Background:**

Sirtuin-7 (SIRT7) is associated with the maintenance of tumorigenesis. However, its functional roles and oncogenic mechanisms in prostate cancer (PCa) are poorly understood. Here, we investigated the roles and underlying molecular mechanisms of SIRT7 in PCa cell growth and androgen-induced autophagy.

**Methods:**

The LNCap and 22Rv1 PCa cell lines were subjected to quantitative reverse transcription (RT)-PCR to characterize their genes encoding SIRT7, AR, and SMAD4. The proteins produced from these genes were quantified by western blotting and immunoprecipitation analysis. SIRT7-depleted cells were produced by transfection with plasmid vectors bearing short hairpin RNAs against SIRT7. The proliferation of each cell line was assessed by CCK8 and EdU assays. Autophagic flux was tracked by mRFP–GFP–LC3 adenovirus under an immunofluorescence microscope. Apoptosis was evaluated by flow cytometry. Tumors were induced in mouse axillae by injection of the cell lines into mice. Tumor morphology was examined by immunohistochemistry and relative tumor growth and metastases were compared by a bioluminescence-based in vivo imaging system.

**Results:**

SIRT7 depletion significantly inhibited cell proliferation, androgen-induced autophagy, and invasion in LNCap and 22Rv1 cells (in vitro) and mouse xenograft tumors induced by injection of these cells (in vivo). SIRT7 knockdown also increased the sensitivity of PCa cells to radiation. Immunohistochemical analysis of 93 specimens and bioinformatic analysis revealed that SIRT7 expression was positively associated with androgen receptor (AR). Moreover, the AR signal pathway participated in SIRT7-mediated regulation of PCa cell proliferation, autophagy, and invasion. SIRT7 depletion downregulated the AR signal pathway by upregulating the level of SMAD4 protein in PCa cells.

**Conclusion:**

SIRT7 plays an important role in the development and progression of human PCa and may be a promising prognostic marker for prostate cancer.

## Background

Prostate cancer (PCa) is prevalent and remains the second leading cause of cancer death among males [1]. In 2018, there were new 1,276,106 cases of PCa, accounting for 13.5% of all male tumors [2]. It has been reported that androgen plays a vital role in the growth, invasion, and progression of PCa [3, 4]. Androgen induces transcriptional activation of the androgen receptor (AR), which is a member of the steroid receptor family. Androgen receptor can be modulated by coregulators that upregulate (coactivators) or downregulate (corepressors) it and its target genes [5]. Androgen deprivation therapy (ADT), surgical castration, chemotherapy, radiation, or a combination of these approaches are standard treatments for advanced prostate cancer. However, most cases of PCa become castration-resistant (CRPC) within 18–36 months after androgen deprivation therapy, which are currently incurable. Several resistance mechanisms are driven largely by AR signaling [6–8]. In addition, androgen can induce autophagy and autophagic flux of PCa cells via the AR pathway to promote cell proliferation [9, 10]. Although androgens and AR are involved in the progression and functioning of PCa, the mechanisms responsible for these activities are unclear. Therefore, studies are needed to determine the mechanism underlying the AR-mediated regulation of PCa to predict the occurrence of PCa and develop new treatments for this disease.

SIRT7 belongs to the sirtuin family (SIRT1–7) of mammalian NAD^+^-dependent deacetylases. They are localized in different subcellular compartments, share a highly conserved catalytic core domain, and target various substrates. The roles of SIRT7 in various tumors have been proposed but remain controversial [11–14]. Recently, two studies reported the effects of SIRT7 in PCa [15, 16]. They found that elevated SIRT7 levels enhance tumor aggressiveness by promoting epithelial-to-mesenchymal transition (EMT). In human non-small cell lung cancer, depletion of SIRT7 can inhibit gemcitabine-induced autophagy and significantly sensitize cancer cells to gemcitabine therapy in vivo and in vitro [17]. However, the mechanism by which SIRT7 influences PCa proliferation and androgen-induced autophagy is unclear. Recent studies reported that SIRT7 can decrease the protein level of SMAD4 by deacetylating and destabilizing SMAD4 protein [13, 18]. SMAD4 is an important AR corepressor which can inhibit the transcription of AR [19]. The effect of SMAD3 (an AR coactivator) on AR transcription, also depends on SMAD4 expression [20, 21]. Therefore, we speculate that SIRT7 may regulate PCa cell proliferation and autophagy via SMAD4-mediated AR signaling.

We examined SIRT7-free prostate cells to identify the roles and mechanisms of this factor in PCa. We used human clinical samples and published data to investigate the relationship between SIRT7 and AR expression in PCa. We established that SIRT7 promoted prostate cancer proliferation, autophagy and metastasis via the AR signaling pathway indirectly. SIRT7 depletion increased the SMAD4 protein level and regulated the AR signal in PCa. These findings demonstrated a role for SIRT7 in regulating the AR signaling pathway and androgen-induced autophagy in PCa and highlight the potential of targeting pathways for novel therapeutics.

## Methods

### Cell culture and retroviral infection

The LNCap, 22Rv1, Du145 and PC3 cell lines were obtained from the Cell Bank of the Chinese Academy of Sciences (Shanghai, China). LNCap and 22Rv1 cells were cultured in RPMI 1640 with 10% (w/v) fetal bovine serum (FBS; Gibco, Grand Island, NY, USA) and Du145 and PC3 cells were cultured in DMEM with 10% FBS at 37 °C in a 5% CO_2_ humidified incubator. A lentiviral short hairpin RNA (shRNA) construct targeting SIRT7 (target sequence:5′-GAAGAAGGCAGCCACAGTCGG-3′) was synthesized by Shanghai GenePharma (Shanghai, China). The shRNAs were cloned into the pLKO.1 plasmids (Sigma-Aldrich Corp, St. Louis, MO, USA). For retroviral packaging, 8 μg of *SIRT7* knockdown or control constructs, 8 μg of pSPAX2, and 4 μg of pMD2G were co-transfected into HEK293T cells with Lipofectamine™3000 (Invitrogen, Carlsbad, CA, USA). The supernatant was harvested at 48–60 h after transfection and filtered through a 0.44-μm membrane (Millipore, Billerica, MA, USA). Polybrene (6 μg mL^− 1^) was added to the filtrate. After 48–60 h, virus-infected cells were selected with 2 μg mL^− 1^ puromycin for another 72 h. The SMAD4 siRNAs, ARwt, and SIRT7wt were purchased from GenePharma (Shanghai, China) and transfected into the cells with Lipofectamine™3000 (Invitrogen) according to the manufacturer’s instructions.

### Total RNA extraction and qRT-PCR

Total RNA was extracted from the cells with TRIzol reagent (Takara, Shiga, Japan), reverse-transcribed with PrimeScript™ RT Master Mix (Takara), and subjected to qRT-PCR with SYBR® Premix Ex Taq™ (Takara) according to the manufacturer’s instructions. Relative mRNA expression was calculated by the 2^-ΔΔCt^ method. The PCR primers are listed in Additional file 5: Table S1.

### Western blotting and immunoprecipitation

Cells were lysed in RIPA buffer (Beyotime, Suzhou, China). The lysates were centrifuged at 12,000×*g* and 4 °C for 15 min. The protein samples were quantified by bicinchoninic acid assay (Beyotime). Equal amounts of the protein samples were separated by sodium dodecyl sulfate-polyacrylamide gel electrophoresis (SDS-PAGE) and transferred onto polyvinylidene fluoride (PVDF) membranes (Millipore, Billerica, MA, USA). The membranes were blocked with a 5% (w/v) bovine serum albumin (BSA) solution in TBST (Tris-buffered saline with 0.1% (v/v) Tween 20) at 20–25 °C for 1 h. The membranes were incubated at 4 °C with the primary antibodies rabbit monoclonal anti-AR, PSA (Abcam, Cambridge, UK), anti-matrix metallopeptidase (MMP)-2, anti-MMP-9, anti-Vimentin, anti-Slug, anti-SMAD3, and anti-GAPDH (Cell Signaling Technology, Danvers, MA, USA) as well as anti-SIRT7, anti-ERα and anti-ERβ (Abclonal, Wuhan, China). Acetylation protein probed with pan anti acetyl. After 12 h, the membranes were washed > 3× with phosphate-buffered saline (PBS)-Tween 20 and incubated with horseradish peroxidase (HRP)-conjugated secondary antibodies (Santa Cruz Biotechnology, Dallas, TX, USA) at 20–25 °C for 2 h. The positive protein bands were visualized by enhanced chemiluminescence (ECL) staining (Millipore) and evaluated with an ECL detection system (Millipore). For immunoprecipitation, the cells were lysed in Pierce IP lysis/wash buffer supplemented with protease inhibitors (Thermo Fisher Scientific, Waltham, MA, USA). Cell debris was removed by centrifugation at 13,000×*g* and 4 °C for 15 min. Clear cell lysates were combined with anti-SIRT7 (Abcam, Cambridge, UK) and anti-SMAD4 antibodies or control IgG and incubated at 4 °C overnight. Immunoprecipitates bound to magnetic beads were washed in Pierce IP lysis/wash buffer, eluted in elution buffer (Thermo), and analyzed by western blotting.

### Transmission electron microscopy

To assess autolysosome formation, after steroid starvation for 48 h, the cells were treated with dihydrotestosterone (DHT) for 3 days, and then washed twice with serum-free media. The cells were gently scraped, centrifuged and then fixed for 1 h at room temperature with 4% glutaraldehyde in cacodylate buffer (pH 7.0). Pellets were then embedded and sectioned for TEM analysis at 200 kV. Ultrathin sections were examined on a CM-120 electron microscope (Philips, Eindhoven, Netherlands).

### CCK8 and EdU cell proliferation assays

Unless otherwise noted, the cells were steroid starved for 48 h in phenol red-free medium containing 10% charcoal stripped-FBS and then 1 nM DHT was added to restore androgens to physiological levels. Cell proliferation was assessed with a CCK8 assay kit (Dojindo Laboratories, Kumamoto, Japan). The cells were seeded onto 96-well plates at a density of 2 × 10^3^ well^− 1^ and incubated in a humidified 5% CO_2_ incubator for 24, 48, 72, 96, or 120 h. Next, 10 μL of CCK8 reagent was added to each well and the plates were returned to the incubator for another 2 h. Absorbances was measured at 450 nm in a multiplate reader (BioTek, Winooski, VT, USA). Proliferation of the transfected cells was assessed with a Cell-Light EdU DNA cell proliferation kit (Ribo, Guangzhou, China) according to the manufacturer’s protocol. Transfected cells were incubated for 2 h at 37 °C in culture media supplemented with the thymidine analog ethynyldeoxyuridine (EdU), and then fixed in 4% (v/v) paraformaldehyde (PFA) for 30 min. The cells were permeabilized with 0.5% (v/v) Triton X-100. Next, 1 × Apollo reaction cocktail was added and the cells were incubated for 30 min. EdU incorporation into genomic DNA was visualized under a Leica DMi8 fluorescence microscope (Wetzlar, Germany). Five fields were randomly selected and the percentage of EdU-positive cells was determined.

### Clonogenic assay

In the colony assay, 400 cells of 22RV1 and 600 cells of LNCap per 3 mL medium containing 1 nM DHT were incubated in a 6-well cell culture plate (Corning) for 21 days. The cells were washed with PBS 3 times and fixed for 15 min with 4% (v/v) PFA. The cells were stained with 0.1% (w/v) crystal violet at 20–25 °C for 20 min and washed three times with water. A cell colony was defined as an aggregation with more than 50 cells. The images were detected with a ChemiLucent ECL Detection system (BD) and quantitated with ImageJ software (National Institutes of Health). Each assay was performed in triplicate and the data are shown as the means ± SD. *P*-values were calculated by *t*-test (*P < 0.05; **P < 0.01; ***P < 0.001).

### mRFP–GFP–LC3 adenovirus infection

Autophagic flux was tracked by mRFP–GFP–LC3 adenovirus (Hanbio, Shanghai, China). Forty-eight hours after adenovirus infection, the cells expressed LC3 protein labeled with mRFP–GFP. Yellow puncta (mRFP^+^ and GFP ^+^) correspond to the presence of autophagosomes, and red puncta (mRFP^+^ and GFP^−^) indicate autolysosomes. Red LC3 puncta accumulation was quantified to evaluate autophagic flux. After steroid starvation for 48 h in phenol red-free medium containing 10% charcoal stripped-FBS, the cells were cultured with 0, 1 or 10 nM DHT for 3 days and then imaged under a Leica DMi8 fluorescence microscope. At least 16 cells in each group were analyzed.

### Apoptosis detection

Apoptosis was evaluated with an Annexin-V/PI kit (BD Biosciences, San Diego, CA, USA). The cells were washed with PBS and incubated with AnnexinV/PI at 20–25 °C for 25 min. The cell apoptosis were measured with a fluorescence-activated, cell-sorting Accuri C6 flow cytometer (BD Biosciences).

### Migration, and invasion assays

Cell migration and invasion assays were performed in a Transwell chamber (Corning, Corning, NY, USA) according to the manufacturer’s protocol. For the invasion assays, the Transwell inserts were coated with 15 μg μL^− 1^ Matrigel (Corning). After transfection, 1 × 10^5^ cells were seeded into the upper chamber, which contained 200 μL of serum-free medium. The lower chamber contained 600 μL complete cell culture media. The cells were incubated at 37 °C for 48 h. The cells in the upper chamber were removed with cotton swabs. Those able to pass through the filter were fixed with 4% (v/v) PFA and stained with 0.1% (w/v) crystal violet at 20–25 °C for 20 min. The cells in six randomly selected fields were counted and photographed under an inverted microscope (Leica Microsystems). The assay was performed in triplicate and repeated at least once.

### Tumor xenograft and metastasis experiments

Twelve nude mice (male, 4 weeks, weight 16 ± 2 g) were randomly divided into two groups of six animals each. The 22Rv1 cells were suspended in PBS at a density of 1 × 10^7^/ml. Matrigel (Corning) was added to the cell suspension at a 1:1 ratio, and 150 μL of this mixture was subcutaneously injected into the axillae to induce tumor growth. Every 7 days, the tumors were measured with a caliper. Tumor volumes were calculated as follows: V_tumor_ = 0.5 × L × W^2^, where L = length and W = width. After 4 weeks, the mice were sacrificed and their tumor sizes and weights were measured. In a separate experiment, equal numbers of luciferase-expressing 22Rv1 cells (2 × 10^6^/mL) and control or SIRT7-depleted 22Rv1 cells were injected into mouse tail veins. After 21 days, tumor metastasis was visualized with a bioluminescence-based IVIS (in vivo imaging system) (Caliper Life Sciences, Waltham, MA, USA).

### Immunohistochemistry

Tumor samples were collected, fixed in 4% (v/v) PFA (Invitrogen), and dehydrated with over an ethanol concentration gradient. The tumors were embedded in paraffin, sectioned, and immunohistochemically stained with anti-SIRT7 antibody (Abcam), anti-AR (Abcam), antibody anti-Ki67 antibody (Abcam), anti-LC3B antibody (Cell Signaling Technology). We used Allred Score (scores of 0–8) [22] by evaluating proportion of staining (scores of 0–5) and intensity of staining (scores of 0–3) to quantify the expression of SIRT7 in specimens from PCa patients. Tumor cell morphology was examined under a microscope (Leica Microsystems, Wetzlar, Germany).

### Bioinformatics analysis

The Oncomine database (https://www.oncomine.org/resource/login.html) was used to collect information to analyze of the expression of SIRT7. Gene expression profiling interactive analysis (GEPIA, http://gepia.cancer-pku.cn/help.html) and Kaplan-Meier survival curves were applied to analyze the survival and recurrence rate of patients with PCa.

### Statistical analysis

Data are shown as the means ± standard deviation (SD). Statistical analyses were conducted in GraphPad Prism v. 7.01 (GraphPad Software Inc., San Diego, CA, USA). Analysis of variance, *t*-test or χ^2^-test was used to detect differences between groups. *P* < 0.05 was considered as statistically significant.

## Results

### SIRT7 is upregulated in PCa and correlates with poor patient survival

**Fig. 1:**
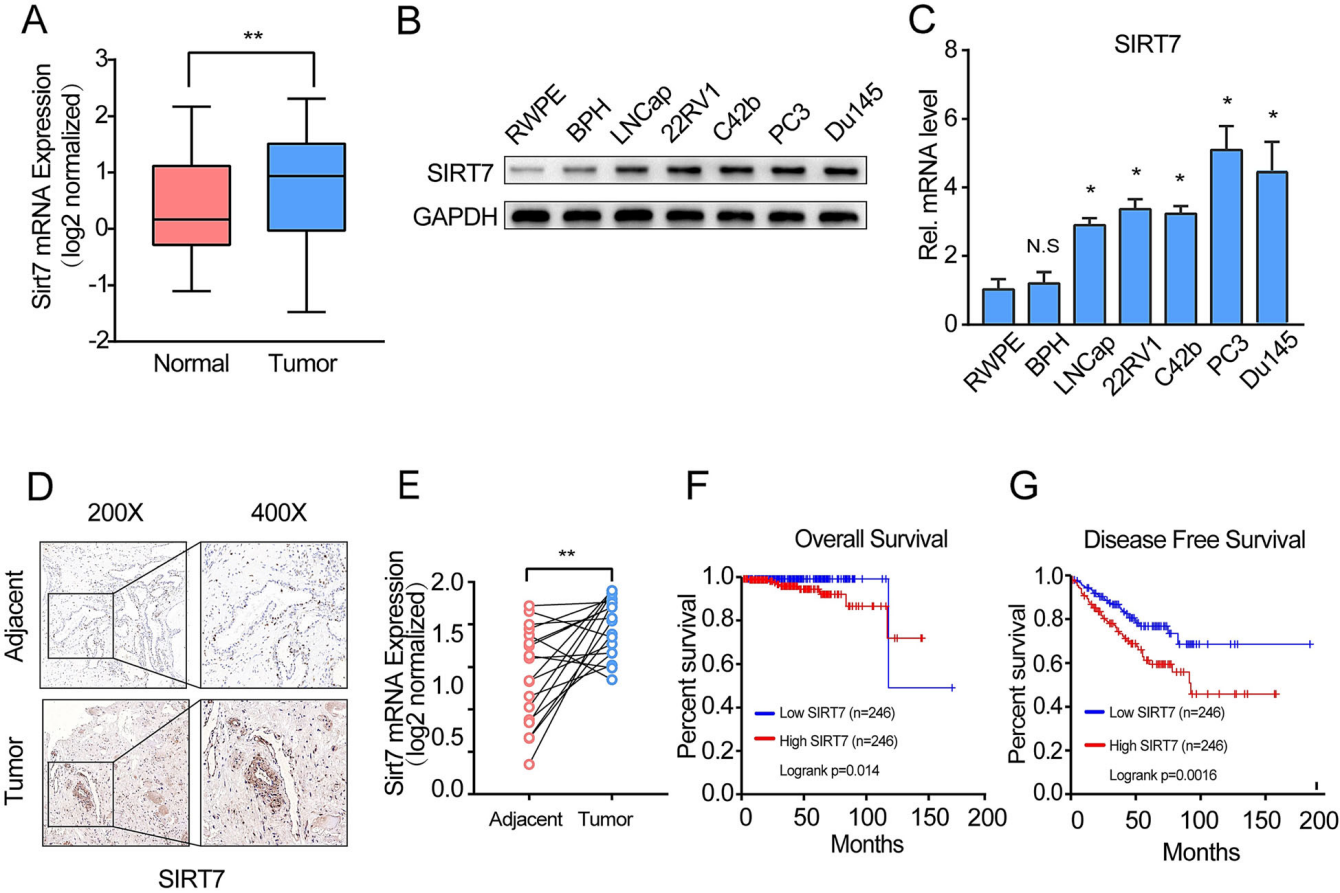
SIRT7 overexpression is associated with poor prognosis in prostate cancer. **a** Meta-analysis of the SIRT7 expression in cancerous prostate tissues relative to that in normal prostate tissues retrieved from a prostate cancer dataset [23–25] in the Oncomine database. **b** Western blotting of SIRT7 expression in normal prostate epithelial and prostate cancer cell lines. **c** RT-PCR of SIRT7 expression in normal prostate epithelial and prostate cancer cell lines. **d** Representative IHC staining of SIRT7 on pairs of prostate tumor and normal adjacent tissues. Scale bar, 50 μm. **e** Analysis of mRNA level of SIRT7 in both prostate cancer and their adjacent normal tissues, retrieved from Wallace et al. [24]. **f** and **g** Kaplan-Meier overall survival and disease-free survival curves for all 572 patients with prostate cancer stratified by high and low SIRT7 expression. Each assay was performed in triplicate and the data are shown as the means ± SD. *P*-values were calculated by *t*-test (*P < 0.05; **P < 0.01; ***P < 0.001)

Whether SIRT7 mRNA expression significantly differs between cancerous and normal prostate tissues is controversial [15, 16]. Thus, we evaluated the SIRT7 expression levels reported in published profiles of patients with PCa [23–25]. SIRT7 was moderately upregulated in cancerous prostate tissue compared to the case in normal prostate tissue (*P* = 0.0057; Fig. 1a). We also measured SIRT7 protein expression in the PCa cell lines LNCaP, 22Rv1, C4-2b, PC3, and DU145, and normal prostate epithelial cell lines RWPE-1 and BPH-1. All five PCa cell lines showed higher SIRT7 protein expression levels than normal prostate epithelial cell lines (Fig. 1b). Real-time PCR confirmed that SIRT7 mRNA was upregulated (by at least 2.9-fold) in cultured cancerous versus normal prostate epithelial cells (Fig. 1c). To eliminate the influence of individual differences among cells, we analyzed SIRT7 protein expression in pairs of PCa tissues. In adjacent normal prostate tissues, SIRT7 was either undetectable or expressed at very low levels. In contrast, SIRT7 protein expression was overexpressed in PCa tissues (Fig. 1d). We further analyzed SIRT7 microarray expression datasets for 18 paired PCa tissues from a dataset [24]. SIRT7 mRNA expression was upregulated in PCa tissues relative to that in their adjacent normal tissues (*P* = 0.0023) (Fig. 1e). Using the web analyses tool of GEPIA (gene expression profiling interactive analysis) [26], Kaplan-Meier survival curves showed that overall survival (OS) in patients with high SIRT7 expression levels was significantly shorter than in those with low SIRT7 expression (*P* = 0.014; Fig. 1e). Moreover, recurrence rates were low in patients with low SIRT7 expression (*P* = 0.002; Fig. 1f). Therefore, SIRT7 was overexpressed at the mRNA and protein levels in PCa cells and tissues, and this was associated with poor OS and disease-free survival (RFS).

### SIRT7 promotes PCa cell proliferation in vitro

**Fig. 2:**
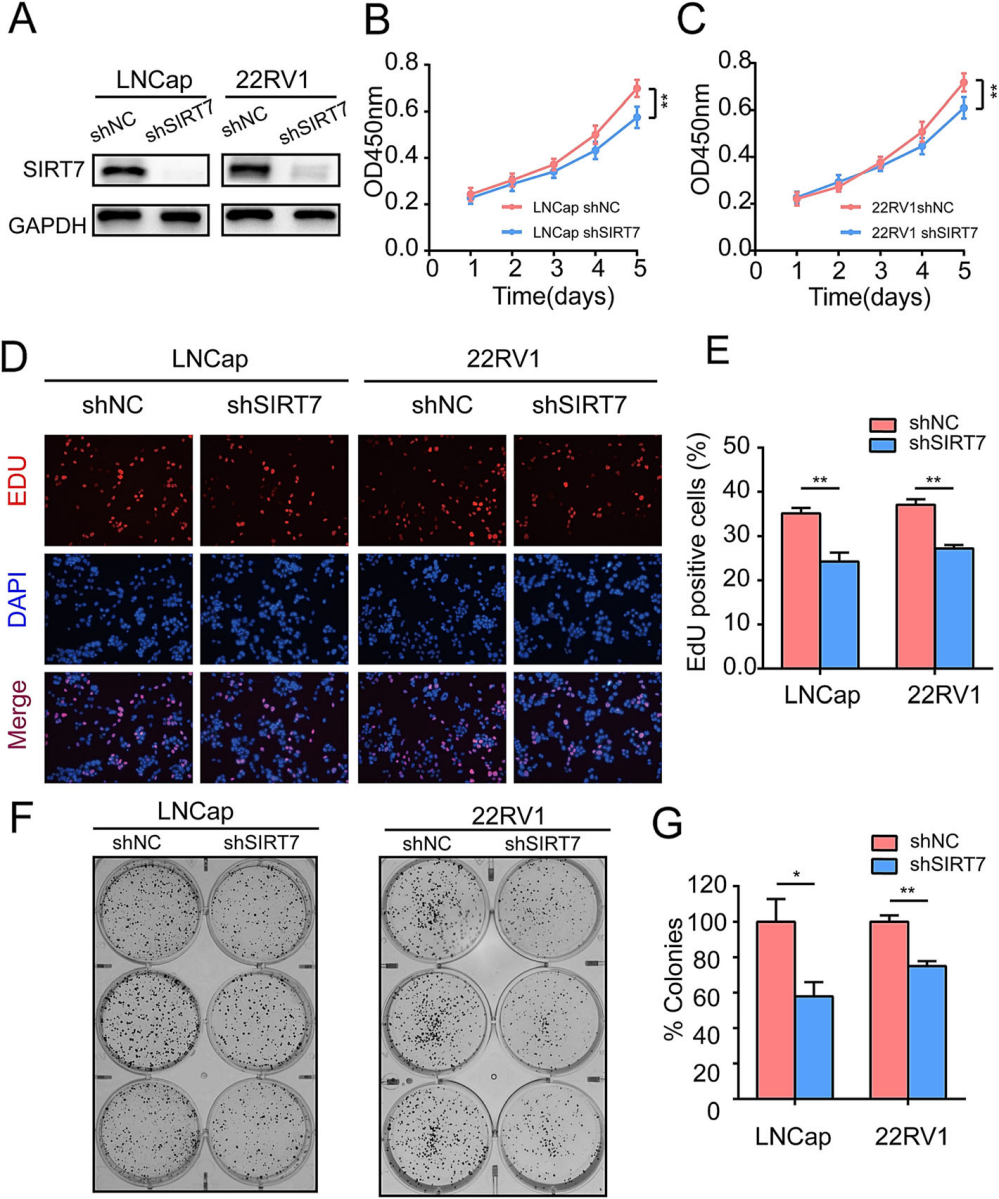
SIRT7 enhances prostate cancer cell growth in vitro. **a** Protein level of SIRT7 in shNC and shSIRT7 groups of LNCap and 22Rv1 cell lines. **b** and **c** CCK8 assay of LNCap (**b**) and 22Rv1 (**c**) from control and SIRT7 knockdown group. **d** Presentative EdU immunofluorescent staining of LNCap and 22Rv1 from control and SIRT7 knockdown group. **e** Percentages of EdU-positive cells of the indicated groups. **f** and **g** Colony formation assay of shNC and shSIRT7 groups in LNCap and 22Rv1 cell lines. Each assay was performed in triplicate and the data are shown as the means ± SD. *P*-values were calculated by *t*-test (*P < 0.05; **P < 0.01; ***P < 0.001)

Previous studies mainly focused on the influence of SIRT7 on PCa metastasis [15, 16]. However, the effects of SIRT7 on PCa cell proliferation are unclear. We ectopically knocked down SIRT7 in LNCap and 22Rv1 PCa cell lines (Fig. 2a). CCK8 assays revealed that SIRT7 depletion substantially inhibited LNCap and 22Rv1 proliferation (Fig. 2b). We also conducted EdU assays to measure SIRT7-deleted prostate cell proliferation. As shown in Fig. 2c and d, 35% of LNCap and 38% of 22Rv1 cells with SIRT7 knockdown incorporated EdU. In contrast, only 21% of LNCap and 28% of 22Rv1 vector-infected control prostate cells incorporated EdU (*P =* 0.0029 and *P =* 0.0010, respectively). We also examined the effect of SIRT7 on the proliferation of LNCap and 22Rv1 cells following the forced expression of SIRT7 and found that the proliferation of both PCa cell lines was promoted by SIRT7. (Additional file 1: Figure S1) The mean colony number (Fig. 2e) was also significantly decreased in LNCap and 22Rv1 cells with SIRT7 depletion compared to vector-transfected PCa control cells (*P* = 0.0126 and *P* = 0.007, respectively).

### SIRT7 modulates PCa cell autophagy, aggressiveness and radiation resistance in vitro

**Fig. 3:**
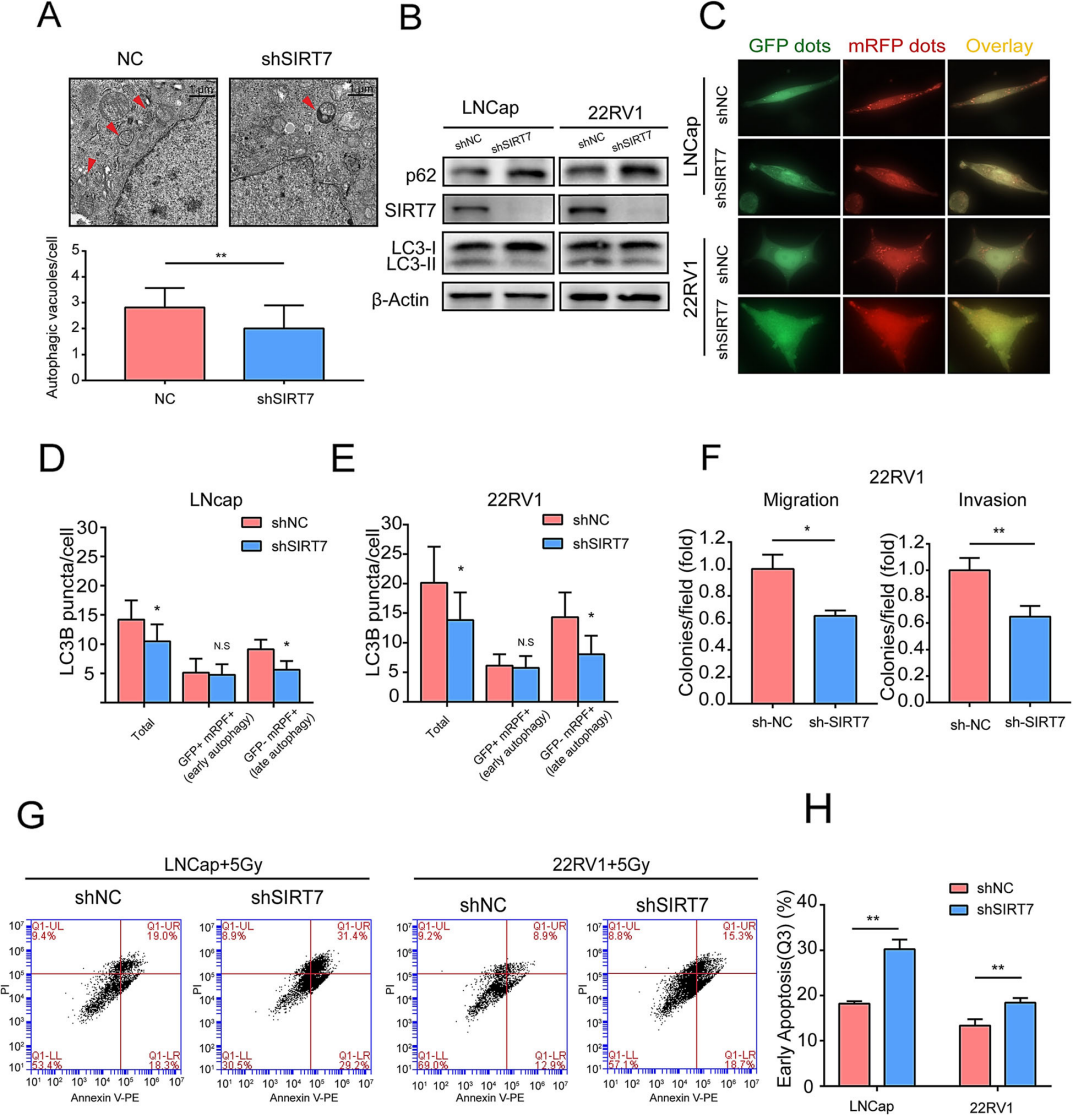
SIRT7 promotes prostate cancer cell autophagy and aggressiveness in vitro. **a** Transmission electron micrographs showing autophagic vacuoles in 22RV1 cells cultured with 1 nM DHT for 3 days before fixation. Arrows indicate autophagosome structures. Scale bar, 1 μm. **b** Western blot analysis to determine LC3BI/II levels of LNCaP and 22RV1 cells treated with vehicle or DHT (1 nM) for 3 days. **c** LNCaP and 22RV1 cells stably expressed the mRFP-GFP-LC3 protein and treated for 3 days with vehicle or DHT (1 nM). Autophagosomes (yellow) and autolysosomes (red) was co-visualized and examined by immunofluorescence microscopy. **d** and **e** Mean number of puncta of each cell (*n* = 16 cells) was analyzed and plotted. **f** Transwell migration and invasion assays showing the effects of SIRT7 on prostate cancer cell migration and invasion. **g** Apoptosis was analyzed via flow cytometry. **h** Percent apoptosis was determined in Q3. Each assay was performed in triplicate and the data are shown as the means ± SD. *P*-values were calculated by *t*-test (*P < 0.05; **P < 0.01; ***P < 0.001)

Because androgen can promote cell proliferation by inducing the autophagy of PCa cells, we investigated whether SIRT7 depletion can inhibit the androgen-mediated autophagy and autophagic flux of PCa cells. We constructed the SIRT7-depleted PCa cell lines 22Rv1 and LNCaP, which were treated for 72 h with 1 nM DHT to assess the formation of autophagosomes and autolysosomes. We compared the effect of SIRT7 depletion on androgen-induced autophagy by transmission electron microscopy. The 22Rv1 cells with SIRT7 depletion showed more autophagosome formation than 22Rv1 cells with transfected vector (*P* = 0.0093), suggesting that SIRT7 modulates androgen-induced autophagy in 22RV1 cells (Fig. 3a). To further assess whether SIRT7 depletion affects androgen-induced autophagy in PCa, we evaluated the conversion of LC3B-I to LC3B-II, an important marker of autophagy. In both SIRT7-depleted LNCap and 22RV1 cells, the conversion of LC3BI to LC3BII was decreased significantly compared to that in wild-type cells (Fig. 3b). We transiently transfected the cells with mRFP-GFP-LC3 and quantified the different LC3 punctate numbers to assess the impact of SIRT7 depletion on autophagic flux in PCa cells. As GFP is sensitive to the acidic environment of the lysosome, yellow punctate (GFP^+^ and mRFP^+^) indicated an early autophagosome, whereas red punctate (GFP^−^ and mRFP^+^) suggested late autophagy which meant LC3 had been delivered to the lysosomes (Fig. 3c). Compared to wild type cells, SIRT7 depletion led to a significant decrease in red punctate (GFP^−^ and mRFP^+^) formation, indicating that SIRT7 depletion inhibited the autophagic flux induced by androgen (Fig. 3d, e).

To determine whether the SIRT7 level affected androgen-dependent human PCa cell aggressiveness, we performed a Transwell migration assay to determine whether SIRT7 depletion influenced PCa cell migration. Compared to wild type cells, SIRT7 depletion significantly decreased LNCap and 22Rv1 migration by 26.9% (*P =* 0.010) and 34.9% (*P =* 0.019), respectively. The Transwell assay revealed that SIRT7 depletion impaired cell invasion through a Matrigel basement membrane matrix. Compared to wild type cells, SIRT7 knockdown inhibited LNCap and 22Rv1 invasion by 24.2% (*P =* 0.009) and 35.1% (*P =* 0.008), respectively (Fig. 3 and Additional file 2: Figure S2 C). Factors regulating epithelial-to-mesenchymal transition (EMT), matrix metalloproteinases (MMPs), and vascular endothelial growth factor (VEGF) participate in tumor metastasis. SIRT7 knockdown in 22Rv1 cells downregulated the mesenchymal marker Vimentin, EMT-inducing transcription factor Slug, MMP2 and MMP9, and VEGF-A (Additional file 2: Figure S2).

As radiation resistance is associated with tumor aggressiveness, we explored the role of SIRT7 in the radiation sensitivity of PCa cells. Exposure to radiation treatment (5 Gy) significantly increased apoptosis in SIRT7 knockdown LNCap and 22RV1 (*P =* 0.0079 and *P =* 0.0087, respectively). Thus, SIRT7 may be involved in tumor radiation resistance (Fig. 3h, i). Based on the results, SIRT7 expression conferred PCa cells with androgen-mediated autophagy, aggressiveness and radiation resistance.

### SIRT7 depletion impairs tumor proliferation, autophagy and metastasis in vivo

**Fig. 4:**
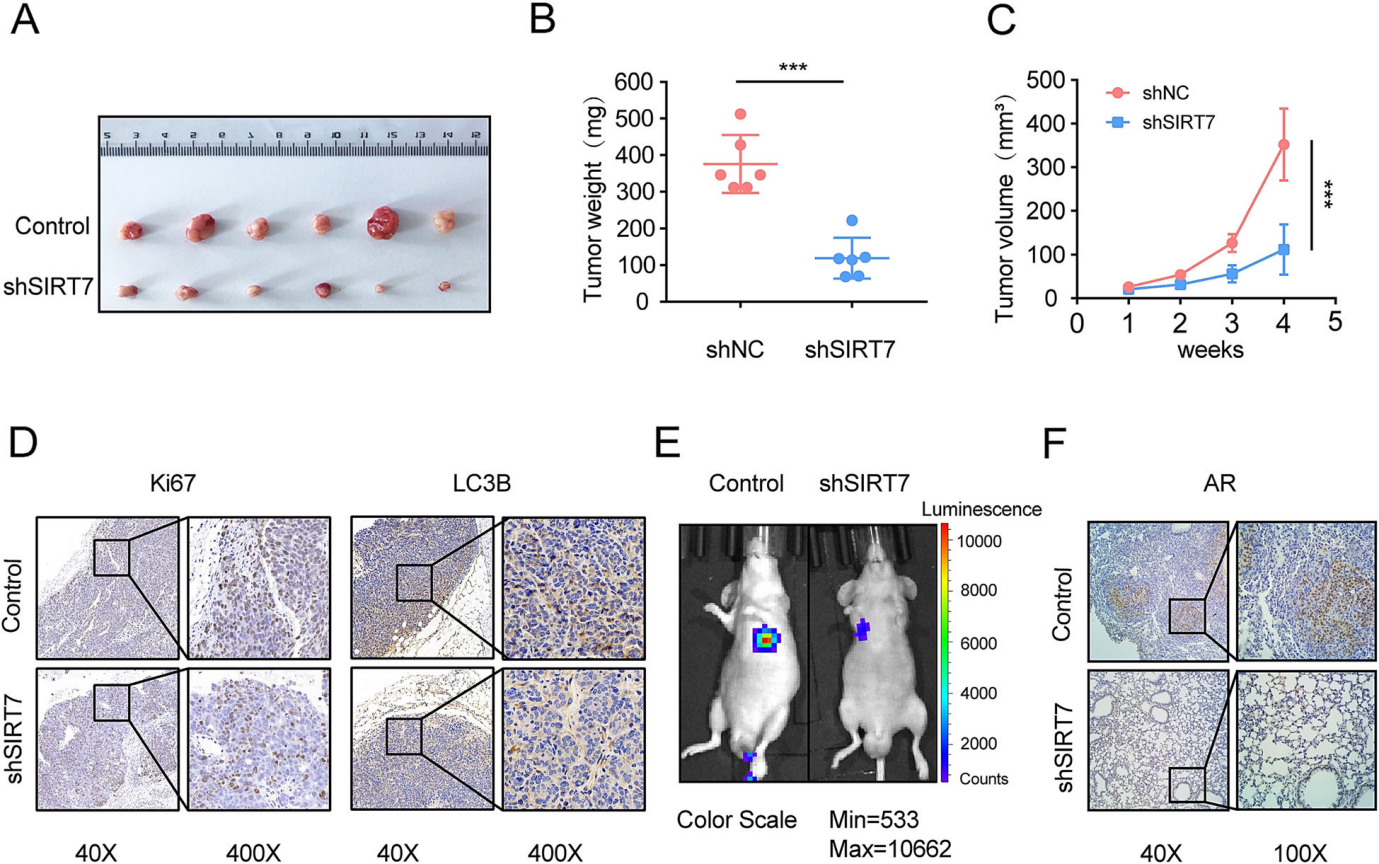
Effects of SIRT7 on tumor xenografts in nude mice. **a** 22RV1 cells with stable SIRT7 silencing by shRNA (shNC and shSIRT7) were injected subcutaneously into the axillae of nude mice. Mice were sacrificed and their tumors were photographed after 28 days. **b** Xenograft tumors were measured every 3 days. V_tumor_ = 0.5 × L × W^2^. **c** Mice were sacrificed after 28 days and their tumor masses were excised and weighed. **d** Xenograft samples were subjected to Ki67 and LC3B staining. Scale bar, 50 μm. **e** 22RV1 cells with stable SIRT7 silencing by shRNA (shNC and shSIRT7) were used in a metastasis model assay induced by tail vein injection. Luminescence of 22RV1 cells was evaluated by an in vivo imaging system. **f** Representative lung metastasis samples were stained with AR. Scale bar, 50 μm. *P*-values were calculated by *t*-test (*P < 0.05; **P < 0.01; ***P < 0.001)

We injected both wild type- and SIRT7-depleted 22Rv1 cells into nude mice and measured the tumor volume and growth after 4 weeks. Tumor weight and volume in animals administered SIRT7-depleted 22Rv1 cells increased significantly but more slowly (*P* = 0.0001 and *P* = 0.0003, respectively) than those in mice injected with normal 22Rv1 cells (Fig. 4a–c). Immunohistochemical (IHC) analysis showed that the animals injected with SIRT7-depleted 22Rv1 cells had a lower percentage proliferation of Ki67^+^ and LC3^+^ tumor cells than mice administered wild type 22Rv1 cells (Fig. 4d). For the metastasis assay, luciferase-expressing 22Rv1 cells and either control or SIRT7 knockdown vectors were injected into mouse tail veins. We evaluated the metastasis of 22Rv1 cells with an in vivo imaging system. Compared to animals injected with control cells, mice administered SIRT7-depleted 22Rv1 cells presented with in vivo PCa metastasis at 21 days post-treatment (Fig. 4e). IHC showed that animals injected with type 22Rv1 cells had more lung metastatic modules (Fig. 4f). Therefore, SIRT7 inactivation in PCa cells impaired their proliferation, autophagy and metastasis in vivo.

### SIRT7 upregulation in prostate cancer tissues correlates with AR signaling

**Fig. 5:**
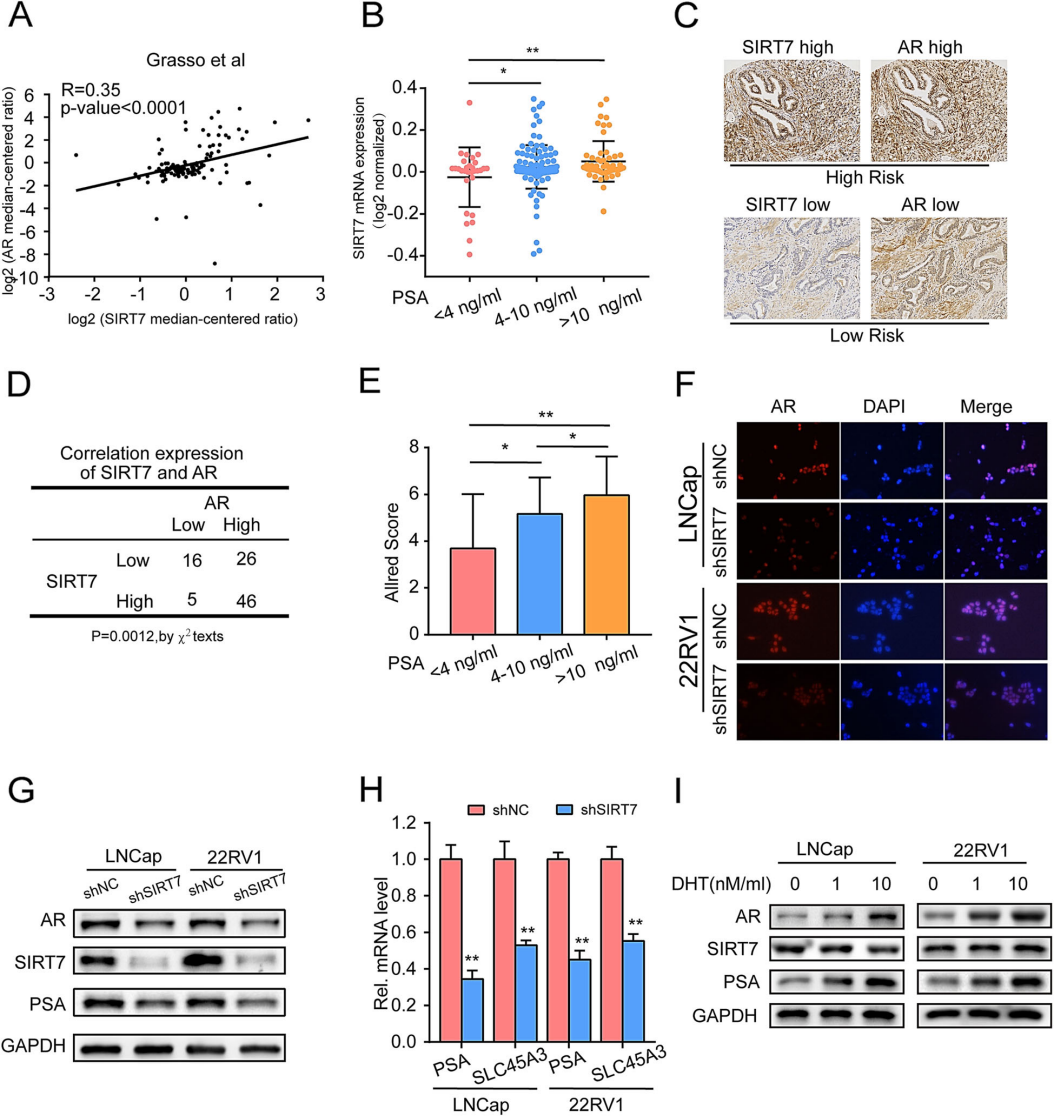
SIRT7 upregulation in prostate cancer tissues correlates with the AR signaling (**a**) Analysis of the correlation between SIRT7 and AR mRNA expression in the prostate cancer dataset [25]. **b** Analysis of the association between SIRT7 and serum PSA expression in the prostate cancer dataset using the Oncomine database [48]. **c** Representative images of a human prostate cancer tissue array showing SIRT7 and AR protein levels in high-risk tumor and low-risk tumor. Scale bar, 50 μm. **d** Overall correlated expression of SIRT7 and AR protein in the human prostate cancer tissue array of (**c**). **e** Association between serum PSA and SIRT7 Allred score in each prostate cancer tissue specimen. **f** Immunofluorescence showed that SIRT7-depletion downregulated AR in prostate cancer cells. **g** Western blot results showing the modulation of the AR and its target genes in SIRT7-depleted prostate cancer cells. **h** RT-qPCR determined the modulation of the AR target genes in SIRT7-depleted prostate cancer cells. **i** Western blotting revealed that SIRT7 expression was not affected by the level of AR expression in prostate cancer cells. Each assay was performed in triplicate and the data are shown as the means ± SD. *P*-values were calculated by *t*-test or χ^2^-test (*P < 0.05; **P < 0.01; ***P < 0.001)

Androgen and AR signaling are closely associated with the development and progression of PCa. Based on this, we investigated the relationship between AR and SIRT7. First, we analyzed the correlation between SIRT7 and AR mRNA using the Oncomine dataset [25] (Fig. 5a). There was a significant correlation between SIRT7 and AR (r = 0.35, *P* < 0.0001). We next verified the relationship between SIRT7 mRNA and pre-treatment serum prostate-specific antigen (PSA) levels, as PSA is an AR target and prognostic factor for PCa. SIRT7 expression was higher in tumors with PSA ≥ 10 ng mL^− 1^ (*P* = 0.005) and PSA = 4–10 ng mL^− 1^ (*P* = 0.035) than in those with PSA < 4 ng mL^− 1^ (Fig. 5b). Next, we examined 93 PCa specimens by IHC analysis to evaluate the correlation between SIRT7 and AR protein levels. The overall clinicopathological parameters are summarized in Table 1. The SIRT7 and AR protein levels in the PCa tissues were determined by IHC microscopy. There was a significant correlation (*P* = 0.0012) between SIRT7 and AR expression (Fig. 5c, d). We applied the Allred score [15, 22] to quantify the expression of SIRT7 and identify the correlation between SIRT7 and serum PSA. The SIRT7 expression showed higher mean Allred scores in tumors with PSA ≥ 10 ng mL^− 1^ (*P* = 0.0018) and PSA = 4–10 ng mL^− 1^ (*P* = 0.0288) than in those with PSA < 4 ng mL^− 1^ (Fig. 5e). Significant differences in Allred scores were also found between tumors with PSA = 4–10 ng mL^− 1^ and PSA ≥ 10 ng mL^− 1^ (*P* = 0.0317). To validate the causal role of AR in vitro, we knocked down SIRT7 in LNCap and 22Rv1 cell lines with AR expression. According to the immunofluorescence assay, AR was significantly downregulated in SIRT7-depleted LNCap and 22Rv1 cells (Fig. 5f). Western blot analysis confirmed that AR and its target genes were significantly downregulated in LNCap and 22Rv1 cells with SIRT7 depletion (Fig. 5g). PSA and SLC45A3 were AR target genes. The qRT-PCR analysis showed that under androgenic conditions (1 nM mL^− 1^ DHT), PSA and SLC45A3 were significantly downregulated in LNCap and 22Rv1 cells (Fig. 5h). To determine whether the activity of AR regulates the SIRT7, we used different concentrations of androgen to culture LNCap and 22RV1 cells for 3 days. Western blotting showed that the activity of AR signaling was upregulated with increasing androgen concentrations. However, there was no significant change in the expression of SIRT7. Thus, SIRT7 expression in PCa affected the expression and activity of AR. Additionally, the LNCap and 22RV1 cell lines contained estrogen receptors alpha (ERα) or estrogen receptors beta (ERβ), which may differentially modulated AR responses [27, 28]. Whether SIRT7 depletion modulated the expression of ERα and ERβ to inhibit AR expression? Thus, we further examined the expression of ERα and ERβ in several PCa cell lines with or without SIRT7 depletion. The results showed that SIRT7 depletion did not significant affect the expression of ERα and ERβ. (Additional file 3: Figure S3A) Considering that LNCaP cells harbored an abnormal AR system and 22Rv1 cells also contained AR variants, we overexpressed wild type AR gene in PC-3 cells (AR lacking). We found that SIRT7 depletion inhibited the expression of wild type AR. However, no obvious autophagy was induced by treatment with androgen and cell proliferation was not inhibited in PC3 or PC-3-AR cells with SIRT7 depletion (Additional file 3: Figure S3C, D).

**Table 1:** Characteristics of the 93 patients with prostate cancer

	N(%)
Patients	93
Age	72.5 (8.9)
PSA(ng/ml)	16.4 (9.8)
<4	17
4–10	42
>10	34
Tumor stage	
T2a	13
T2b	25
T2c	33
T3a	10
T3b	7
T4	5
Gleason score	
Gleason ≤6	32
Gleason 7	21
Gleason ≥8	40

### AR mediates the effects of SIRT7 on androgen-dependent PCa cell proliferation, autophagy, and invasion

**Fig. 6:**
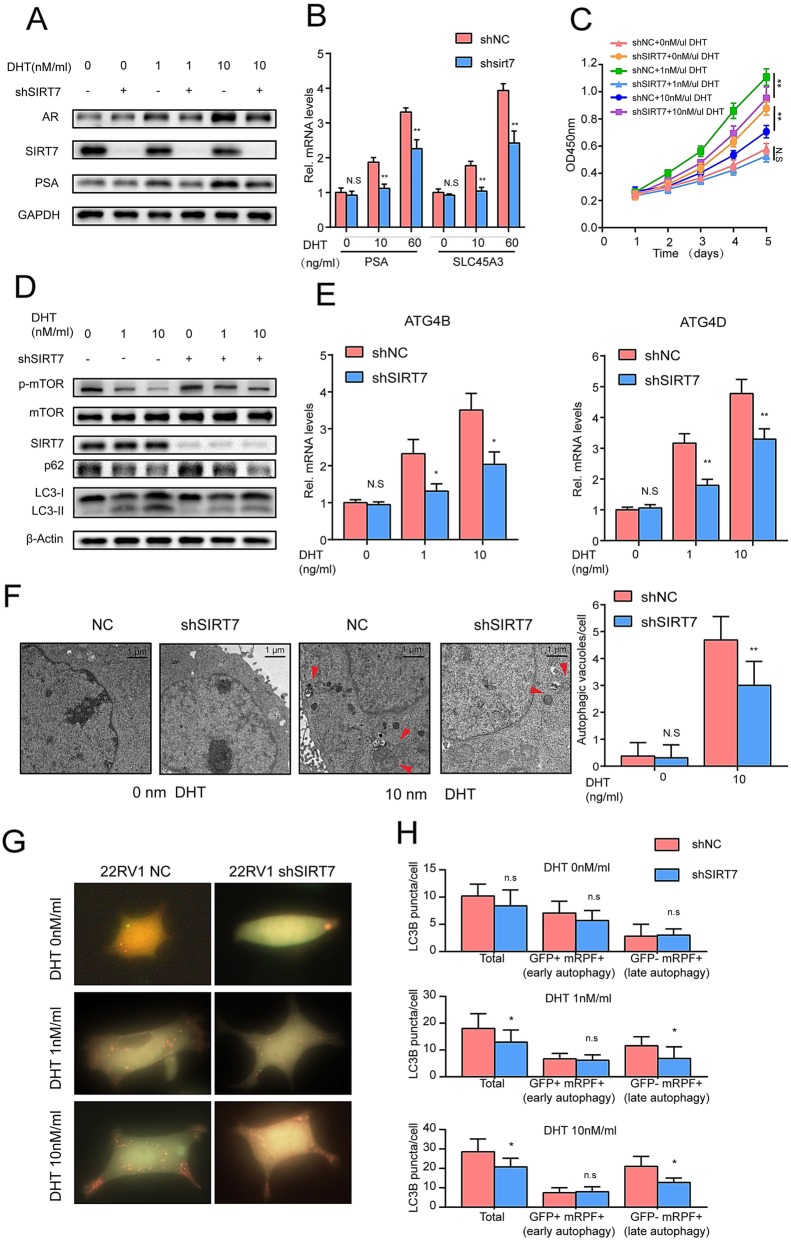
AR mediates the effects of SIRT7 on prostate cancer cell proliferation and androgen-induced autophagy. **a** Western blot determined the modulation of AR in SIRT7-depleted prostate cancer cells cultured in media supplemented with various androgen concentrations (0, 1 and 10 nM DHT). **b** RT-qPCR determined the modulation of PSA and SLC45A3s in SIRT7-depleted cancer cells cultured in media supplemented with various DHT concentrations. **c** CCK8 assay measured the effect of SIRT7 knockdown on prostate cancer cell proliferation in media supplemented with various androgen concentrations. **d** Western blot data showing p-mTOR, mTOR, AR, p62, and SIRT7 expression levels and LC3-II conversion in SIRT7-depleted cancer cells cultured with various DHT concentrations. **e** RT-qPCR revealed the modulation of ATG4B and ATG4D (both are AR-regulated autophagy genes) in SIRT7-depleted cancer cells cultured with various DHT concentrations. **f** Transmission electron micrographs for detecting autophagic vacuoles in 22RV1 cells cultured with 0 and 10 nM DHT for 3 days before fixation. Arrows indicate autophagosome structures. Scale bar, 1 μm. **g** 22RV1 cells stably expressed the mRFP–GFP–LC3 protein and were treated for 3 days with vehicle (ethanol) or DHT. Autophagosomes (*yellow*) and autolysosomes (*red*) were co-visualized and examined by immunofluorescence microscopy. **h** Mean number of puncta of each cells (*n* = 16 cells) treated with various androgen concentrations was analyzed and plotted. Each assay was performed in triplicate and the data are shown as the means ± SD. *P*-values were calculated by *t*-test (*P < 0.05; **P < 0.01; ***P < 0.001)

As AR signaling is closely related to prostate proliferation, autophagy and migration in PCa, we next investigated the AR-mediated effects of SIRT7 on the proliferation and androgen-induced autophagy of PCa cells. We cultured 22Rv1 cells in medium containing 10% (w/v) charcoal-stripped-FBS supplemented with 0, 1 or 10 nM DHT. Relative to the case in wild type 22Rv1 cells, AR was downregulated in SIRT7-depletion 22Rv1 cells in the presence of 1 and 10 nM DHT but not in cells without DHT (Fig. 6a). We performed RT-PCR to verify the changes in PSA and SLC45A3 mRNA and evaluated the activity of AR under various DHT concentrations. At 1 and 10 nM DHT, the PSA and SLC45A3 mRNA levels in SIRT7-depleted 22Rv1 cells were significantly lower than those in wild type 22Rv1 cells. In contrast, there were no significant differences in the PSA and SLC45A3 mRNA levels between SIRT7-knockdown and wild type 22Rv1 cells in the absences of DHT (Fig. 6b). The CCK8 assays showed that SIRT7 knockdown substantially impaired 22Rv1 cell proliferation in the presence of androgen (Fig. 6c). In contrast, the SIRT7 knockdown and wild type 22Rv1 cell numbers did not significantly differ in the absence of DHT.

We assessed LC3BII levels in wild type and SIRT7-depletied 22RV1 cells treated with various androgen concentrations. Compared to wild type 22Rv1 cells, the LC3BII levels in SIRT7-depleted 22Rv1 cells were decreased significantly in the presence of 1 and 10 nM DHT but not in those without DHT (Fig. 6d). We also conducted RT-PCR to verify the changes in ATG4B and ATG4D, which are two important autophagy genes and could be regulated by AR [9, 10]. There were no significant differences in ATG4B and ATG4D mRNA levels between SIRT7-knockdown and wild type 22Rv1 cells in the absences of DHT. However, the mRNA levels of these two genes were significantly lower in SIRT7 knockdown 22Rv1 cells than in wild type 22Rv1 cells with 1 and 10 nM DHT (Fig. 6e). We tracked autophagosome in normal 22RV1 cells and 22RV1 with SIRT7 depletion by TEM. Compared to wild type 22RV1 cells, SIRT7-depletion led to a significant decrease in autophagosome in the presence of 10 nM DHT but not in those without DHT (Fig. 6f). We further assessed autophagic flux by performing transient transfection of mRFP-GFP-LC3 and then quantified the LC3 punctate numbers. Compared to wild type cells, SIRT7-depletion led to a significant decrease in late autophagy (GFP^−^ and mRFP^+^) in the presence of 1 and 10 nM DHT but not in the absence of DHT (Fig. 6g, h). These results indicate that SIRT7-depletion impaired androgen-induced autophagy by regulating AR signaling.

**Fig. 7:**
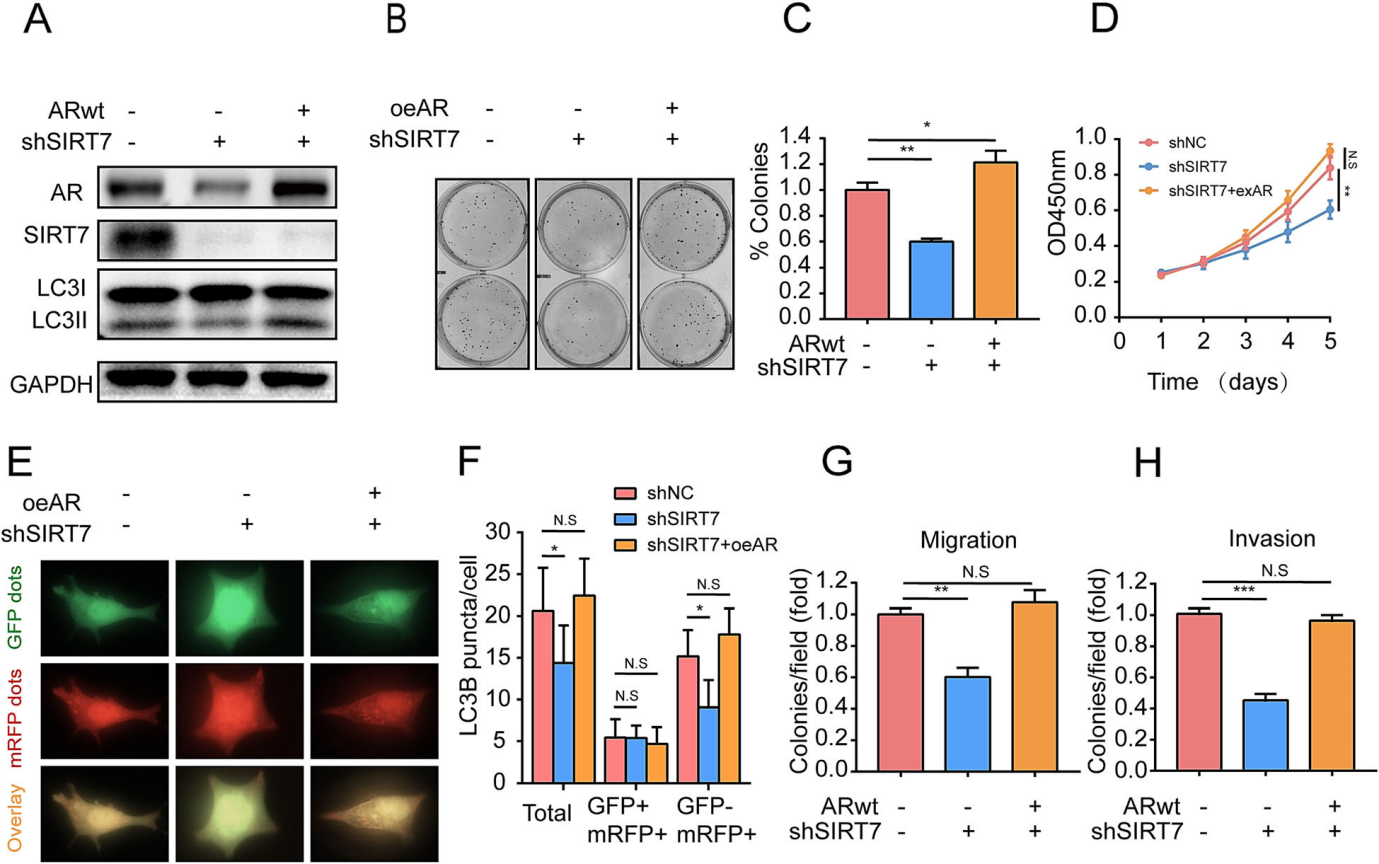
Restored AR expression can restore effects of SIRT7 on prostate cancer cells proliferation, autophagy and aggressiveness. **a** SIRT7 knockdown by shRNA and AR overexpression by plasmids in 22RV1 cells were validated by western blotting. **b** and **c** Colony formation assay on 22RV1 cells shows that AR upregulation reversed the effect of SIRT7 depletion on prostate cell proliferation. **d** CCK8 assay shows that AR upregulation reversed the effect of SIRT7 depletion on prostate cell proliferation. **e** 22RV1 cells stably expressing the mRFP–GFP–LC3 protein were treated for 3 days with vehicle (ethanol) or DHT. Autophagosomes (*yellow*) and autolysosomes (*red*) were covisualized and examined by immunofluorescence microscopy. **f** Mean number of puncta of each cells (*n* = 16 cells) was analyzed and plotted. **g** and **h** Transwell migration and Matrigel invasion assays in 22RV1 cells show that AR upregulation reversed the effect of SIRT7 depletion on prostate cancer cell migration and invasion. Each assay was performed in triplicate and the data are shown as the means ± SD. *P*-values were calculated by *t*-test (*P < 0.05; **P < 0.01; ***P < 0.001)

To verify whether SIRT7 depletion impaired PCa cell proliferation, autophagy and invasion by altering AR expression, we restored the AR gene in SIRT7-depleted 22Rv1 cells (Fig. 7a). Colony formation and CCK8 assays revealed that the mean colony number was restored in SIRT7-depleted AR-overexpressing 22Rv1 cells, compared to that in SIRT7-depleted 22Rv1 cells without AR upregulation (Fig. 7b–d). The level of LC3BII in western blot assays (Fig. 7a) and number of red punctate (GFP^−^ and mRFP^+^) was restored in SIRT7-depleted AR-overexpressing 22Rv1 cells (Fig. 7e, f). The Transwell migration and invasion assays revealed that impairment of SIRT7-depleted 22Rv1 cell migration and invasion was restored by upregulating AR expression (Fig. 7g, h). These results indicated that upregulation of AR expression opposed the effects of SIRT7 depletion on androgen-dependent PCa cell proliferation, autophagy and invasion.

### SIRT7 promotes AR signal pathway expression via SMAD4

**Fig. 8:**
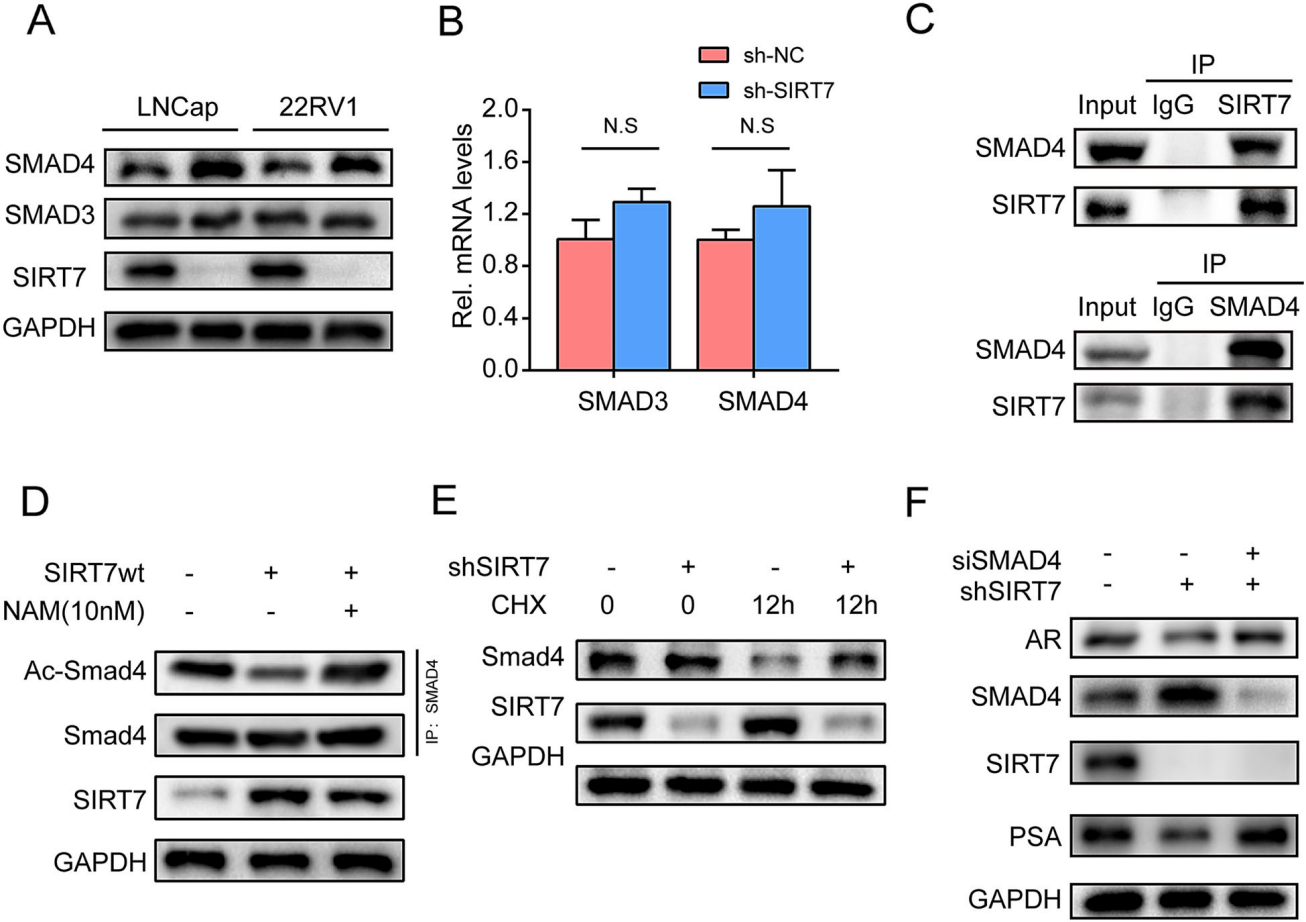
SIRT7 promotes AR expression via SMAD4. **a** Western blotting revealed the SMAD3 and SMAD4 protein levels in SIRT7-deficient prostate cancer cells. **b** RT-qPCR was performed to detect SMAD3 and SMAD4 mRNA levels in SIRT7-deficient prostate cancer cells. **c** Co-immunoprecipitation of endogenous SMAD4 with anti-SIRT7 antibodies in 22RV1 cells. Co-immunoprecipitation of endogenous SIRT7 with anti-SMAD4 antibodies in 22RV1 cells. **d** Immunoblots showing the acetylation levels of SMAD4 in the presence of wild-type SIRT7 and nicotinamide (NAM). Acetylatied proteins were immunoprecipitated with anti-SMAD4 and probed with pan anti acetyl antibodies. **e** Immunoblots showing SMAD4 levels in the presence of CHX, with or without SIRT7 depletion. **f** Western blot shows that SMAD4 downregulation by siRNA reversed the effect of SIRT7 on AR activity. Each assay was performed in triplicate and the data are shown as the means ± SD. *P*-values were calculated by *t*-test (*P < 0.05; **P < 0.01; ***P < 0.001)

We attempted to determine the mechanism by which SIRT7 regulates the AR signal pathway. We hypothesized that SIRT7 physically interacts with AR. However, SIRT7 was absent in anti-myc-AR precipitates and AR was not detected in anti-FLAG-SIRT7 immunoprecipitates (Additional file 4: Figure S4 A,B). Moreover, no interaction between endogenous SIRT7 and AR was detected (Additional file 4: Figure S4C). We then predicted that SIRT7 controls AR via a regulator. SMAD4 inhibits the AR signal as an important coregulator. We determined whether SIRT7 knockdown regulates SMAD4 protein and mRNA expression in PCa cells. In 22Rv1 cells, SIRT7 knockdown upregulated SMAD4 protein but had no effect on SMAD3 protein (Fig. 8a). RT-PCR verified that the SMAD3 and SMAD4 mRNA levels were unchanged in SIRT7-knockdown PCa cells (Fig. 8b). We verified the interaction between endogenous SIRT7 and SMAD4 in 22Rv1 cells. SIRT7 was detected in anti-SMAD4 immunoprecipitates and SMAD4 was found in anti-SIRT7 immunoprecipitates (Fig. 8c). To confirm that SIRT7 decreases the acetylation level of SMAD4 in PCa, we transfected wild-type SIRT7 into 22Rv1 cells and detected the acetylation level of SMAD4. Cells with SIRT7 showed lower acetylation level of SMAD4. In addition, the reduction in acetylation was abolished by inhibiting SIRT7 with nicotinamide (NAM) (Fig. 8d). Next, we used cycloheximide (CHX) to inhibit protein synthesis and the degradation of SMAD4 was detected. Compared to that in 22RV1 cells with SIRT7 depletion, more SMAD4 protein was degraded in wide type 22Rv1 cells (Fig. 8e). To confirm that SIRT7 influences the AR signaling pathway by affecting SMAD4, we knocked down SMAD4 in SIRT7-depleted 22Rv1 cells. RNAi to deplete SMAD4 abolished repression of the AR signal by SIRT7 knockdown. The expression of PSA, a downstream target of AR, was also restored (Fig. 8e).

## Discussion

SIRT7 is a type of NAD^+^-dependent deacetylase. It mediates normal and cancerous cellular activity by regulating various signal pathways and targeting its protein substrate [29]. SIRT7 is highly expressed in numerous tumors including PCa, which is associated with aggressive PCa phenotypes [15, 16]. However, its precise function in PCa is unknown. Here, we demonstrated that SIRT7 is a key oncogene promoting prostate tumorigenesis. SIRT7 is significantly upregulated in PCa and its level is correlated with those of AR and PSA. SIRT7 depletion inhibited proliferation, colony formation, androgen-mediated autophagy, and promoted radiation sensitivity. SIRT7 depletion also upregulated SMAD4, which controls the AR signal in PCa.

Elevated SIRT7 expression is observed in breast cancer [12], thyroid tumorigenesis [11], and colon cancer [14]. However, the involvement of SIRT7 expression in PCa, however, is controversial. Haider et al. [15] reported that SIRT7 mRNA expression is constant in both normal and cancerous prostate glands. Both normal prostate epithelial (P69) and PCa cell lines show the same SIRT7 protein expression levels. However, Barber et al. [16] reported that SIRT7 mRNA and protein were significantly overexpressed in human prostate tumor samples compared to that in normal prostate gland tissues. The results of the present study are consistent with those of Barber et al. SIRT7 mRNA and protein are upregulated in PCa cell lines compared to those in normal prostate epithelial RWPE-1 and BPH-1 cells. In addition, the protein expression of SIRT7 in PC3 and DU145 did not correlated with the relative mRNA levels. As reported previously, compared to healthy glands specimens, the expression of SIRT7 mRNA in some tumor specimens was not significantly increased but the expression of protein was higher [15]. These results suggest that the increase in SIRT7 protein expression occurs because of a post transcriptional regulation. These discrepancies may also be explained by cell type-specific mechanisms and differences among specimens, cultivation method and detection methods.

The exact function of SIRT7 in tumorigenesis remains unclear. SIRT7 may promote oncogenesis, tumor growth, and metastasis in gastric cancer [14] and hepatocellular carcinoma [30]. In contrast, it can inhibit head and neck squamous cell carcinoma progression [18] and breast cancer metastasis [13]. These discrepant results regarding the effect of SIRT7 on oncogenesis are attributed to potential tumor-specific discrepancy. PCa invasion and migration were significantly decreased after SIRT7 knockdown, which is consistent with those of previous studies [15, 16]. SIRT7 knockdown also affected cancer cell proliferation. Multiple signaling pathways are involved in PCa including PI3K/Akt, NF-κB, TGF-β, Wnt/β-catenin, and AR signaling pathways [31–34]. In addition, there is some crosstalk between AR and the above pathways in PCa. In the presence of testosterone or DHT, AR is translocated to the nucleus via the nuclear pore complex where it affects target genes and influences PCa cell behaviors. Blocking of genetic or pharmacological AR signals downregulates MMP-2 and MMP-9 and reduces the invasiveness of androgen-dependent and androgen-independent PCa cell lines. As a target gene of AR, PSA is mainly regulated by an upstream promoter and enhancer androgen response element [35]. A rapidly rising PSA indicates activation of AR signaling pathways [36] and predicts the poor prognosis [37]. As androgen-independent PCa cell lines, DU145 and PC3 are much more invasive than LNCaP and 22Rv1 cells. However, neither DU145 nor PC3 expresses AR or PSA. Thus, it is not suitable to use PC3 or DU145 cells to study whether SIRT7 affects cancer cell proliferation and invasion by influencing the AR signaling pathway. Here, we used LNCaP and 22Rv1 cancer cells expressing endogenous AR and PSA to examine the relationship between AR and SIRT7 and the effects of the latter on AR signaling-mediated cancer proliferation and aggressiveness. SIRT7 expression increased with AR and the level of serum PSA. SIRT7 depletion inhibited AR signaling activity in androgen-dependent PCa cells. We also examined the effect of SIRT7 depletion on proliferation and androgen-induced autophagy in PC3 cells. The proliferation of PC3 cells with SIRT7 depletion did not change significantly. Androgen treatment for 3 days did not induce autophagy of wild type PC3 and PC3 with SIRT7 depletion (Additional file 3: Figure S3 B, C), suggesting that androgen-induced autophagy was caused by AR-mediated increases in reactive oxygen species and AR-regulated autophagy genes [9, 10]. These results also suggest that SIRT7 depletion affected the proliferation of androgen-dependent PCa cells by reducing androgen-induced autophagy. Based on these results, we predicted that SIRT7 regulates AR signal pathway activity in PCa. Nevertheless, we found no direct molecular interaction between SIRT7 and AR (Additional file 4: Figure S4). Therefore, SIRT7 may indirectly activate AR signaling.

Autophagy is a cellular process that occurs in all eukaryotic cells. This process is associated with various pathologies, including cancer, infection and aging [38, 39]. Autophagy can degrade and remove unnecessary cellular components and promote cellular remodeling for carrying out specialized functions. Recent studies have shown that autophagy can help cancer cells survive under harsh conditions by improving the survival ability of cancer cells under low oxygen, lack of nutrition, chemotherapy and so on. Yan et al. demonstrated that androgen can promote PCa cell proliferation by increasing autophagy and autophagic flux via the AR pathway [10]. Androgen promotes autophagy by increasing reactive oxygen species and upregulating the transcription of autophagy genes *ATG4B, ATG4D, ULK1*, and *ULK2* which are also AR-regulated genes [9]. In our study, SIRT7 depletion impaired the proliferation, autophagy and autophagic flux induced by androgen, and inhibited the expression of ATG4B and ATG4D. These results suggest that SIRT7 inhibited androgen-induced autophagy and proliferation by affecting AR signaling activity. In human non-small cell lung cancer cells, SIRT7 depletion inhibited autophagy and promoted gemcitabine-induced cell death [17]. In PCa, SIRT7 depletion can enhance the sensitivity to docetaxel [15]. In our study, we also found that SIRT7 depletion could enhance the sensitivity to radiotherapy in PCa. This result may be due to the fact that SIRT7 depletion can inhibit cell autophagy and impair cell survival ability under harsh conditions.

Considering that LNCaP cells and 22Rv1 cells contained an abnormal AR system or AR variants which may differentially modulate the wide type AR, we overexpressed the wild type AR gene in PC-3 cells (AR lacking). In our study, androgen-induced autophagy was not observed in either PC3-AR or PC3-AR cells with SIRT7 depletion. This demonstrates that the cell response to androgen might be different, depending on the AR status and cell type [40]. Androgen-induced autophagy was activated when androgen promoted cell growth [10]. However, androgen did not promote cell proliferation in PC3 or PC3-AR cells. We found that AR expression was decreased in PC3-AR cells with SIRT7 depletion, indicating that SIRT7 also modulates wild type AR. ERα or ERβ can modulate the proliferation and migration in PCa [41, 42]. Androgen stimulates the association of the AR and ERβ with Src and activates the Src/Raf-1/Erk-2 pathway to improve the proliferation of LNCaP cells [27]. Considering that ERα or ERβ could differentially modulate AR responses [28], we further examined the expression of ERα and ERβ in several PCa cell lines with or without SIRT7 depletion. In our study, SIRT7 depletion did not significantly affect on the expression of ERα and ERβ in PCa cell lines (Additional file 3: Figure S3A). This suggests that SIRT7 may not affect AR by regulating estrogen receptor.

In breast cancer, SIRT7 could significantly downregulate SMAD4 protein by deacetylating and destabilizing SMAD4 protein without affecting its mRNA level [13]. We observed an interaction between SIRT7 and SMAD4 in PCa cells. The mRNA expression of SMAD4 remained unchanged in SIRT7-depleted 22Rv1 cells, whereas SMAD4 protein was upregulated significantly with increased levels of acetylated SMAD4. These results were consistent with those of the study of oral squamous cell carcinoma [18]. We observed that the acetylation level of SMAD4 was decreased in 22RV1 cells with SIRT7 rather than in 22RV1 cells treated with NAM which could inhibit SIRT7 [22] (Fig. 8d). This suggests that SIRT7 can deacetylate SMAD4. Additionally, the degradation of SMAD4 was decreased in SIRT7-depleted 22Rv1 cells when CHX was used to inhibit protein synthesis. Similarly, the reduction of SMAD4 after adding CHX was abolished when SIRT7 was inhibited by NAM or replaced with enzyme-dead SIRT7-H187Y [13]. These results suggest that decreased protein degradation was caused by SIRT7-mediated deacetylation.

The AR and its coregulators were major oncogenic drivers of PCa. Multiple coregulators such as SMAD3, and SMAD4 control AR activation. Previous studies reported that the MH2 domain of SMAD proteins plays an important role in regulating nuclear hormone receptors [19, 28]. R-SMAD and SMAD4 complexes are co-transported into the nucleus and recruit cofactors to regulate the expression of target genes. It has been demonstrated that AR and SMAD4 interact with each other, and androgen promotes the binding of AR and SMAD4. SMAD4 interacts with AR ligand binding domains by its MH2 domain in the C-terminal. The MH2 domain of SMAD4 also exerted the function on the repression of AR transactivation [19]. In addition, the balance between SMAD3 (coactivator) and SMAD4 (corepressor), together with other unknown AR coregulators, may play important roles in AR transactivation. Increased SMAD 4 protein leads to greater levels of hetero-oligomerization between SMAD4 and SMAD3 (repression of AR transactivation) and lower levels of homo-oligomerization between SMAD3 (enhancement or repression of AR), resulting in the regulation of AR transactivation [43, 44]. In our study, we observed that SMAD4 knockdown restored AR expression in 22Rv1 cells with SIRT7 depletion. These results suggest that SIRT7 may regulate AR by regulating SMAD4.

## Conclusions

The present study showed the in vivo and in vitro effects of SIRT7 on PCa. Moreover, SIRT7 is positively correlated with AR and PSA expression. SIRT7 indirectly decreases AR activation and expression via SMAD4. This promotes androgen-induced autophagy, cell growth and metastasis in PCa. Thus, targeting of SIRT7 is promising as a novel therapeutic approach for PCa. These findings suggest that SIRT7 can be used as a prognostic factor for PCa.

## Supplementary information

**Additional file 1: Figure S1.** SIRT7 enhances prostate cancer cell growth in vitro. (A) Protein level of SIRT7 in NC and wild-type SIRT7 groups of LNCap and CCK8 assay of LNCap. (B) Protein level of SIRT7 in NC and wild-type SIRT7 groups of 22RV1 and CCK8 assay of 22RV1. (C) Presentative EdU immunofluorescent staining of LNCap and 22RV1 from control and wild-type SIRT7 groups. (D) Percentages of EdU-positive cells of the indicated groups. Each assay was performed in triplicate and the data are shown as the means ± SD. *P*-values were calculated by *t*-test (*P < 0.05; **P < 0.01; ***P < 0.001).

**Additional file 2: Figure S2.** SIRT7-depletion inhibited the aggressiveness of LNCap and 22RV1 in vitro*.* (A) Western blot results showing the protein levels of Vimentin, Slug, MMP2, and MMP9 in SIRT7-deficient prostate cancer cells. (B) RT-qPCR results showing the modulation of Vimentin, Slug, MMP2, and MMP9 expression in SIRT7-depleted prostate cancer cells. (C) and (D) Transwell migration and invasion assay results showing the effects of SIRT7 on LNCap cell migration and invasion. (E) Western blotting results showing Slug protein levels in SIRT7-deficient LNCap cells. (F) RT-qPCR results showing the modulation of Slug and VEGF-A expression in SIRT7-depleted LNCap cells. Each assay was performed in triplicate and the data are shown as the means ± SD. *P*-values were calculated by *t*-test (*P < 0.05; **P < 0.01; ***P < 0.001).

**Additional file 3: Figure S3.** The effect of SIRT7 in PC3 and PC3-AR. (A)Western blotting results showing the ERα and ERβ in SIRT7-depleted prostate cancer cells. (B) Western blotting results showing the modulation of AR in SIRT7-depleted PC3 and PC3-AR. (C) Western blot analysis revealed LC3BI/II levels of PC3 and PC3-AR cells treated with vehicle or DHT (1 nM) for 3 days. (D) CCK8 assay results of PC3 and PC3-AR cells with or without SIRT7 depletion. (E) Presentative EdU immunofluorescent staining results of PC3 and PC3-AR from control and SIRT7-depletion groups. (D) Percentages of EdU-positive cells in the indicated groups. Each assay was performed in triplicate and the data are shown as the means ± SD. *P*-values were calculated by *t*-test (*P < 0.05; **P < 0.01; ***P < 0.001).

**Additional file 4: Figure S4.** SIRT7 does not physically interact with AR. (A) Immunoblots showed that Myc-AR did not immunoprecipitate with anti-FLAG-SIRT7. (B) Immunoblots showed that FLAG-SIRT7 did not immunoprecipitate with anti-Myc-AR. (C) No endogenous AR was detected in an attempt to co-immunoprecipitate AR with anti-SIRT7 antibodies in 22RV1 cells. (D) No endogenous SIRT7 was detected in an attempt to co-immunoprecipitate SIRT7 with anti-SMAD4.

**Additional file 5: Table S1.** Rt-qPCR primer sequences.

## Data Availability

Please contact author for data requests.

## **A**bbreviations

AR: androgen receptor
BCA: bicinchoninic acid
BPH: benign prostatic hypertrophy
BSA: bovine serum albumin
CCK8: cell counting kit-8
cFBS: charcoal-stripped fetal bovine serum
CRPC: castration-resistant prostate cancer
DHT: dihydrotestosterone
ECL: enhanced chemiluminescence
EdU: ethynyldeoxyuridine
EMT: epithelial-to-mesenchymal transition
FBS: fetal bovine serum
GEPIA: gene expression profiling interactive analysis
IgG: immunoglobin G
IVIS: in vivo imaging system
MAPK: mitogen-activated protein kinase
MMP: matrix metalloproteinase
NF-κB: nuclear factor kappa B
OS: overall survival
PBS: phosphate-buffered saline
PCa: prostate cancer
PFA: paraformaldehyde
PSA: prostate-specific antigen
PVDF: polyvinylidene fluoride
qRT-PCR: quantitative real-time polymerase chain reaction
RPMI: Roswell Park Memorial Institute
SDS-PAGE: sodium dodecyl sulfate-polyacrylamide gel electrophoresis
SIRT7: sirtuin-7
TBS: Tris-buffered saline
TBST: Tris-buffered saline with Tween 20
TGF-β: transforming growth factor beta
VEGF: vascular endothelial growth factor
