# Supplementary figures and images for: SIRT7 depletion inhibits cell proliferation and androgen-induced autophagy by suppressing the AR signaling in prostate cancer
Source: J Exp Clin Cancer Res. 2020 Feb 4;39:28. doi: 10.1186/s13046-019-1516-1 (PMC6998106; doi:10.1186/s13046-019-1516-1)

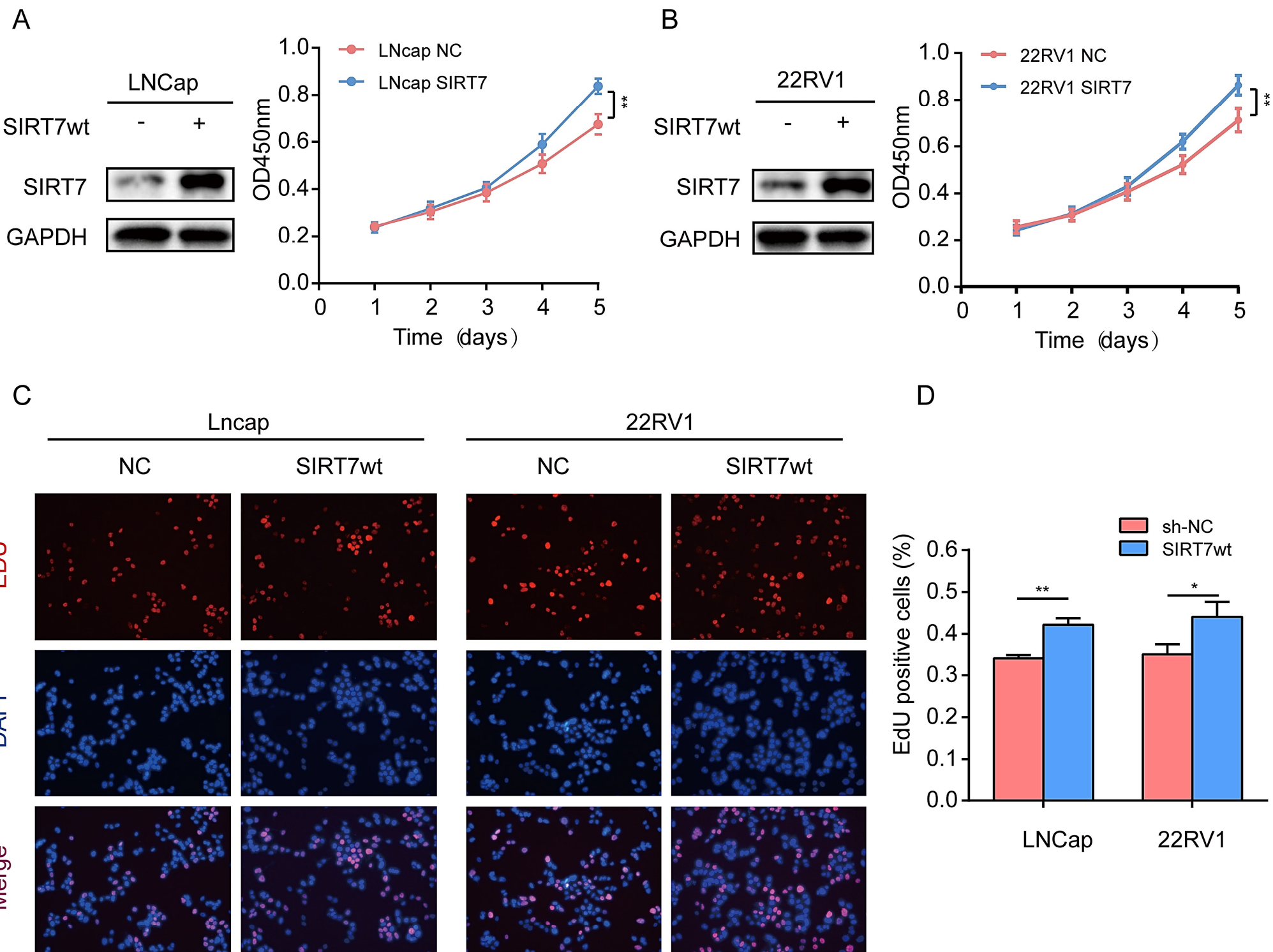

Supplement: Supplementary file 1 — Additional file 1: Figure S1. SIRT7 enhances prostate cancer cell growth in vitro. (A) Protein level of SIRT7 in NC and wild-type SIRT7 groups of LNCap and CCK8 assay of LNCap. (B) Protein level of SIRT7 in NC and wild-type SIRT7 groups of 22RV1 and CCK8 assay of 22RV1. (C) Presentative EdU immunofluorescent staining of LNCap and 22RV1 from control and wild-type SIRT7 groups. (D) Percentages of EdU-positive cells of the indicated groups. Each assay was performed in triplicate and the data are shown as the means ± SD. P-values were calculated by t-test (*P < 0.05; **P < 0.01; ***P < 0.001). [file 13046_2019_1516_MOESM1_ESM.pdf]

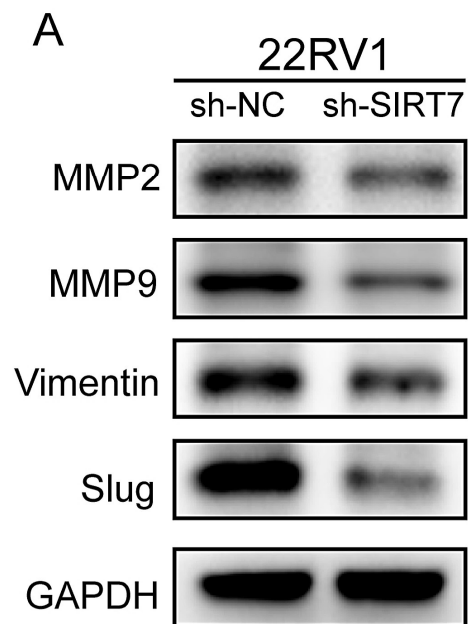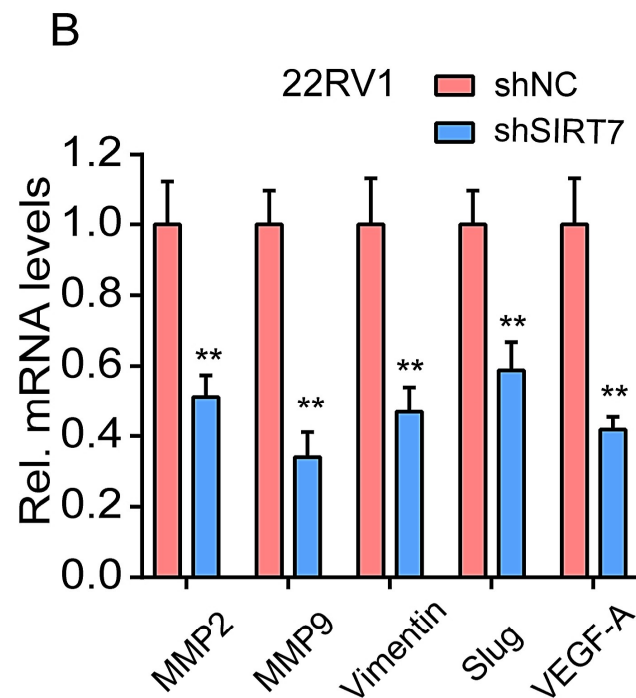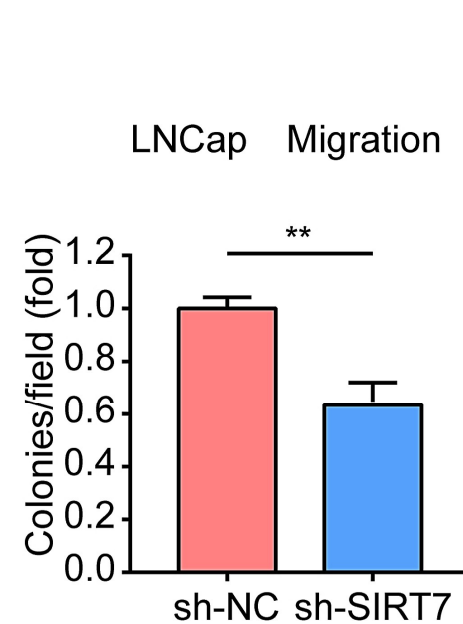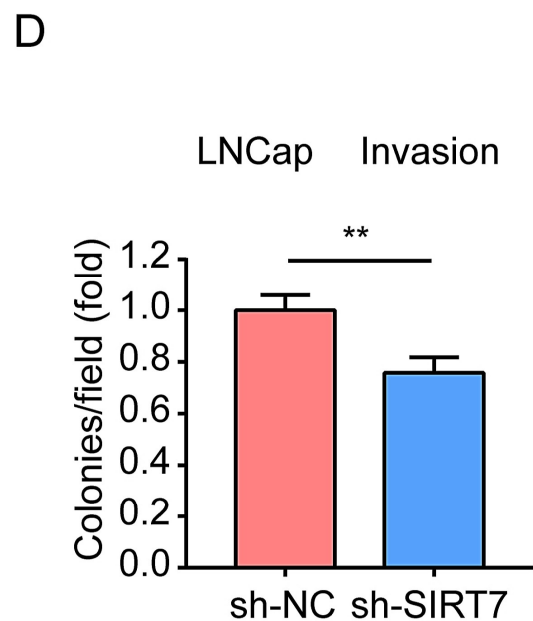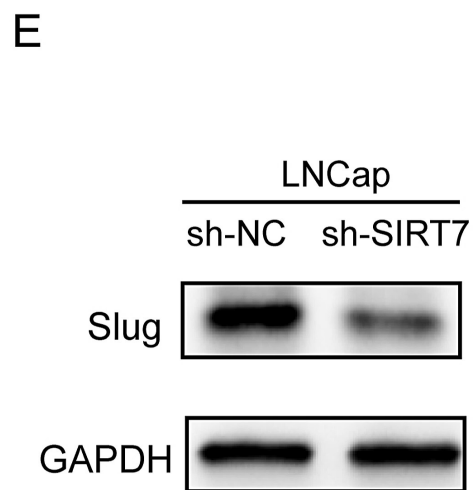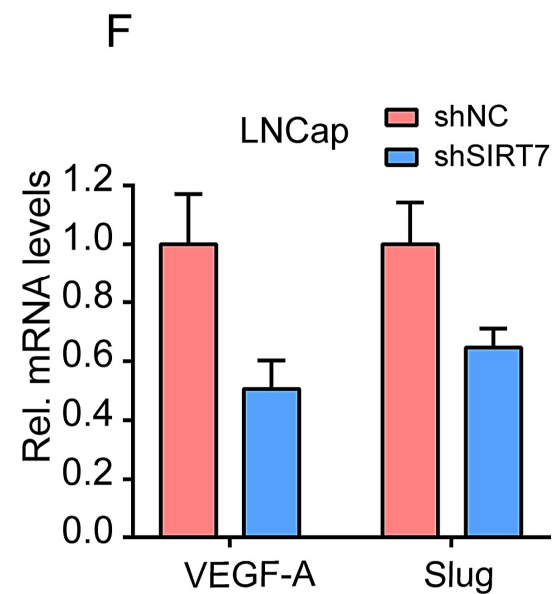

Supplement: Supplementary file 2 — Additional file 2: Figure S2. SIRT7-depletion inhibited the aggressiveness of LNCap and 22RV1 in vitro. (A) Western blot results showing the protein levels of Vimentin, Slug, MMP2, and MMP9 in SIRT7-deficient prostate cancer cells. (B) RT-qPCR results showing the modulation of Vimentin, Slug, MMP2, and MMP9 expression in SIRT7-depleted prostate cancer cells. (C) and (D) Transwell migration and invasion assay results showing the effects of SIRT7 on LNCap cell migration and invasion. (E) Western blotting results showing Slug protein levels in SIRT7-deficient LNCap cells. (F) RT-qPCR results showing the modulation of Slug and VEGF-A expression in SIRT7-depleted LNCap cells. Each assay was performed in triplicate and the data are shown as the means ± SD. P-values were calculated by t-test (*P < 0.05; **P < 0.01; ***P < 0.001). [file 13046_2019_1516_MOESM2_ESM.pdf]

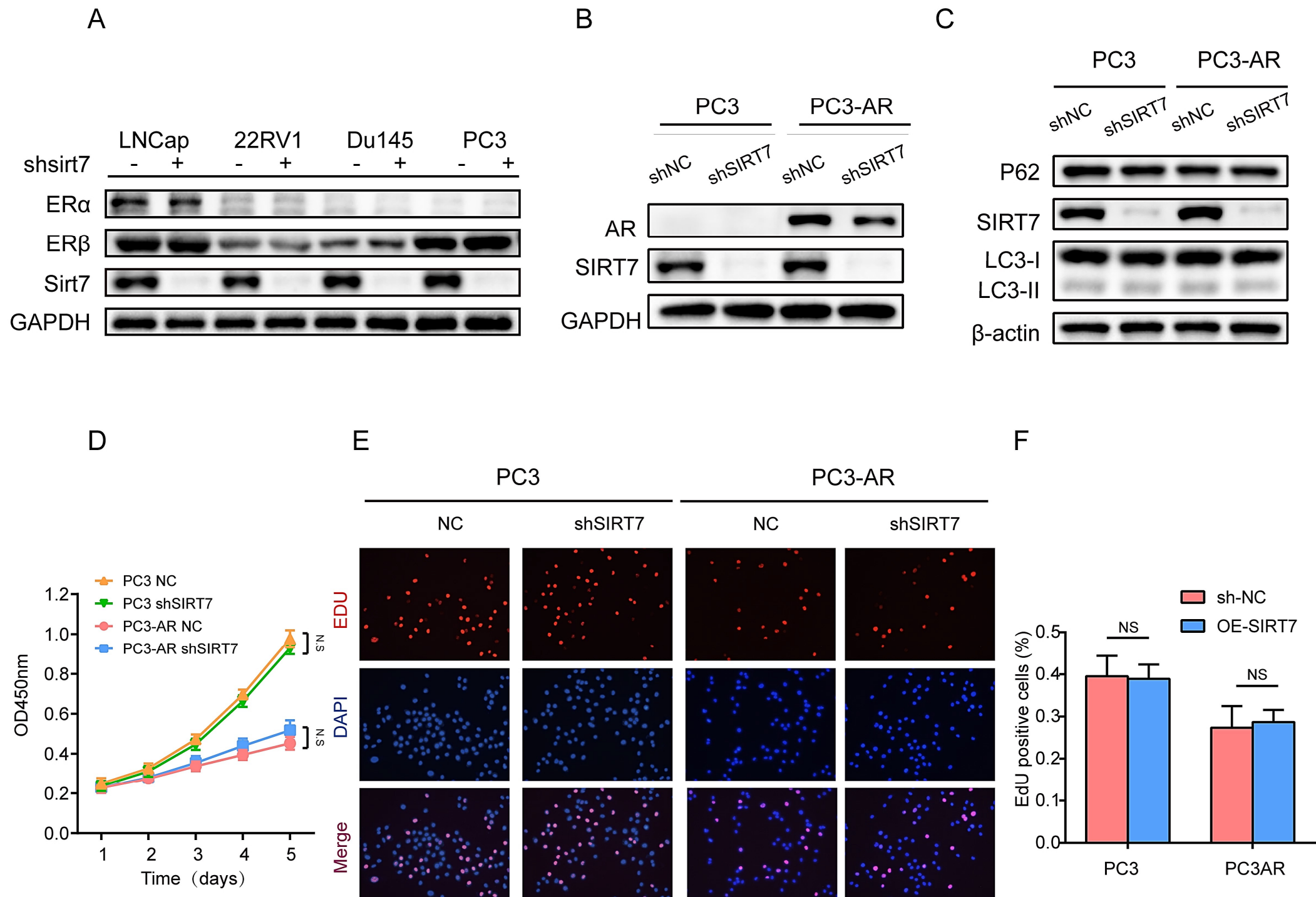

Supplement: Supplementary file 3 — Additional file 3: Figure S3. The effect of SIRT7 in PC3 and PC3-AR. (A)Western blotting results showing the ERα and ERβ in SIRT7-depleted prostate cancer cells. (B) Western blotting results showing the modulation of AR in SIRT7-depleted PC3 and PC3-AR. (C) Western blot analysis revealed LC3BI/II levels of PC3 and PC3-AR cells treated with vehicle or DHT (1 nM) for 3 days. (D) CCK8 assay results of PC3 and PC3-AR cells with or without SIRT7 depletion. (E) Presentative EdU immunofluorescent staining results of PC3 and PC3-AR from control and SIRT7-depletion groups. (D) Percentages of EdU-positive cells in the indicated groups. Each assay was performed in triplicate and the data are shown as the means ± SD. P-values were calculated by t-test (*P < 0.05; **P < 0.01; ***P < 0.001). [file 13046_2019_1516_MOESM3_ESM.pdf]

**A**

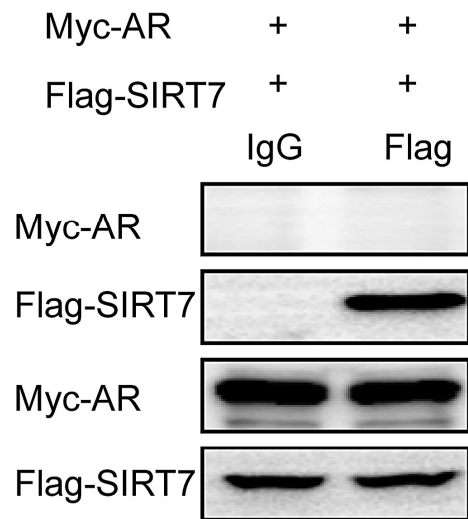

**B**

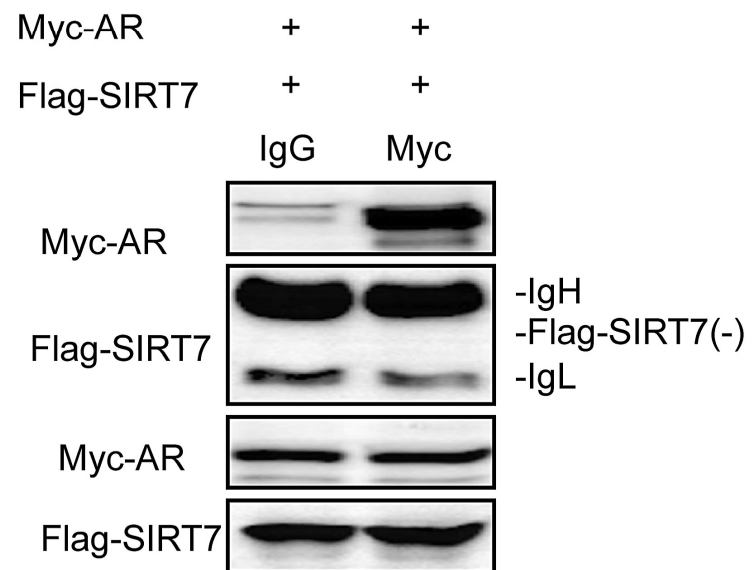

**C**

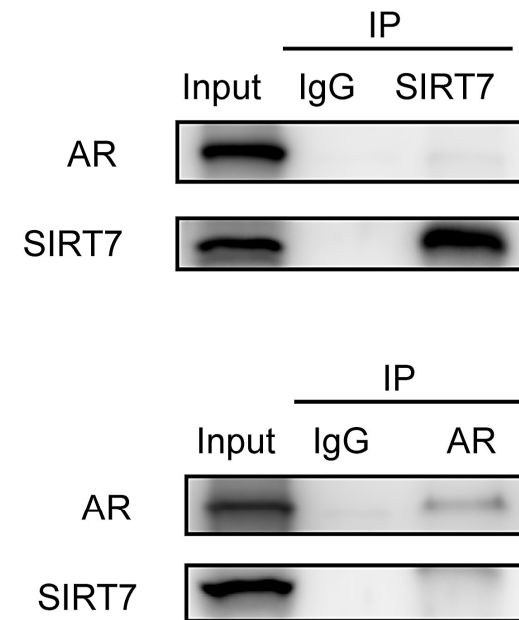

Supplement: Supplementary file 4 — Additional file 4: Figure S4. SIRT7 does not physically interact with AR. (A) Immunoblots showed that Myc-AR did not immunoprecipitate with anti-FLAG-SIRT7. (B) Immunoblots showed that FLAG-SIRT7 did not immunoprecipitate with anti-Myc-AR. (C) No endogenous AR was detected in an attempt to co-immunoprecipitate AR with anti-SIRT7 antibodies in 22RV1 cells. (D) No endogenous SIRT7 was detected in an attempt to co-immunoprecipitate SIRT7 with anti-SMAD4. [file 13046_2019_1516_MOESM4_ESM.pdf]
