# Supplementary material for: SIRT7 depletion inhibits cell proliferation and androgen-induced autophagy by suppressing the AR signaling in prostate cancer
Source: J Exp Clin Cancer Res. 2020 Feb 4;39:28. doi: 10.1186/s13046-019-1516-1 (PMC6998106; doi:10.1186/s13046-019-1516-1)
Supplement: Supplementary file 5 — Additional file 5: Table S1. Rt-qPCR primer sequences. [file 13046_2019_1516_MOESM5_ESM.docx]

Supplementary Table S1. Rt-qPCR primer sequences.

| Primer | Sequence |
| --- | --- |
| PSA Forward | 5′-CACCTGCTCGGGTGATTCTG-3′ |
| PSA Forward | 5′-CCACTTCCGGTAATGCACCA-3 |
| SLC45A3 Forward | 5'- CCTTCACGCTGTTTTACACGG-3' |
| SLC45A3 Reverse | 5'- CGCCTTCATCATAGTGTCTCC -3' |
| SLUG Forward | 5'- TGTTGCAGTGAGGGCAAGAA -3' |
| SLUG Reverse | 5'- GACCCTGGTTGCTTCAAGGA -3' |
| VEGF-A Forward | 5'- CTACCTCCACCATGCCAAGT -3' |
| VEGF-A Reverse | 5'- GCAGTAGCTGCGCTGATAGA -3' |
| ATG4B Forward | 5'-TCGCTGTGGGGTTTTTCTGT-3' |
| ATG4B Reverse | 5'-CACCTCCAAGCAGAGACAGC-3' |
| ATG4D Forward | 5'-GGGCGAGGGTGACATACAG-3' |
| ATG4D Reverse | 5'-ACAGTCCGAGGTCAGGCA-3' |
| MMP2 Forward | 5'-TAGGCCATAGCAGACG -3' |
| MMP2 Reverse | 5'-TTAAGGCCCATATCAGG -3' |
| MMP9 Forward | 5'-ATAGACTACTACAGGCT-3' |
| MMP9 Reverse | 5'-TAGCACGGATAGACCA -3' |
| Vimentin Forward | 5'-GCCCTAGACGAACTGGGTC-3' |
| Vimentin Reverse | 5'-GGCTGCAACTGCCTAATGAG-3' |
| SMAD4 Forward | 5'-AAGCCATTGAGAGAGCAAGGT-3' |
| SMAD4 Reverse | 5'- GGTCACTAAGGCACCTGACC -3' |
| SMAD3 Forward | 5'- TGGACGCAGGTTCTCCAAAC -3' |
| SMAD3 Reverse | 5'- CCGGCTCGCAGTAGGTAAC -3' |
| GAPDH Forward | 5'- AGCCACATCGCTCAGACAC -3' |
| GAPDH Reverse | 5'-CCCTGTCTGTCCTCTGTAGC-3' |
